# The Barriers and Facilitators of eHealth-Based Lifestyle Intervention Programs for People With a Low Socioeconomic Status: Scoping Review

**DOI:** 10.2196/34229

**Published:** 2022-08-24

**Authors:** Isra Al-Dhahir, Thomas Reijnders, Jasper S Faber, Rita J van den Berg-Emons, Veronica R Janssen, Roderik A Kraaijenhagen, Valentijn T Visch, Niels H Chavannes, Andrea W M Evers

**Affiliations:** 1 Faculty of Social and Behavioral Sciences Health, Medical and Neuropsychology Unit Leiden University Leiden Netherlands; 2 Faculty of Industrial Design Engineering Delft University of Technology Delft Netherlands; 3 Department of Rehabilitation Medicine Erasmus University Medical Centre Rotterdam Netherlands; 4 Capri Cardiac Rehabilitation Rotterdam Netherlands; 5 Department of Cardiology Leiden University Medical Center Leiden Netherlands; 6 Vital10 Amsterdam Netherlands; 7 NDDO Institute for Prevention and Early Diagnostics (NIPED) Amsterdam Netherlands; 8 Department of Public Health and Primary Care Leiden University Medical Center Leiden Netherlands; 9 National eHealth Living Lab Leiden University Medical Centre Leiden Netherlands; 10 Medical Delta Leiden University, Delft University of Technology, Erasmus University Delft Netherlands

**Keywords:** eHealth, lifestyle interventions, health behaviors, low socioeconomic status, intervention development, barriers, facilitators, prevention, intervention evaluation

## Abstract

**Background:**

Promoting health behaviors and preventing chronic diseases through a healthy lifestyle among those with a low socioeconomic status (SES) remain major challenges. eHealth interventions are a promising approach to change unhealthy behaviors in this target group.

**Objective:**

This review aims to identify key components, barriers, and facilitators in the development, reach, use, evaluation, and implementation of eHealth lifestyle interventions for people with a low SES. This review provides an overview for researchers and eHealth developers, and can assist in the development of eHealth interventions for people with a low SES.

**Methods:**

We performed a scoping review based on Arksey and O’Malley’s framework. A systematic search was conducted on PubMed, MEDLINE (Ovid), Embase, Web of Science, and the Cochrane Library, using terms related to a combination of the following key constructs: eHealth, lifestyle, low SES, development, reach, use, evaluation, and implementation. There were no restrictions on the date of publication for articles retrieved upon searching the databases.

**Results:**

The search identified 1323 studies, of which 42 met our inclusion criteria. An update of the search led to the inclusion of 17 additional studies. eHealth lifestyle interventions for people with a low SES were often delivered via internet-based methods (eg, websites, email, Facebook, and smartphone apps) and offline methods, such as texting. A minority of the interventions combined eHealth lifestyle interventions with face-to-face or telephone coaching, or wearables (blended care). We identified the use of different behavioral components (eg, social support) and technological components (eg, multimedia) in eHealth lifestyle interventions. Facilitators in the development included iterative design, working with different disciplines, and resonating intervention content with users. Facilitators for intervention reach were use of a personal approach and social network, reminders, and self-monitoring. Nevertheless, barriers, such as technological challenges for developers and limited financial resources, may hinder intervention development. Furthermore, passive recruitment was a barrier to intervention reach. Technical difficulties and the use of self-monitoring devices were common barriers for users of eHealth interventions. Only limited data on barriers and facilitators for intervention implementation and evaluation were available.

**Conclusions:**

While we found large variations among studies regarding key intervention components, and barriers and facilitators, certain factors may be beneficial in building and using eHealth interventions and reaching people with a low SES. Barriers and facilitators offer promising elements that eHealth developers can use as a toolbox to connect eHealth with low SES individuals. Our findings suggest that one-size-fits-all eHealth interventions may be less suitable for people with a low SES. Future research should investigate how to customize eHealth lifestyle interventions to meet the needs of different low SES groups, and should identify the components that enhance their reach, use, and effectiveness.

## Introduction

Chronic diseases, such as cardiovascular disease and type 2 diabetes, accounted for 74% of deaths globally in 2019 [1]. These diseases are often preventable and treatable. Adopting a healthy lifestyle, such as smoking cessation, increased physical activity, a balanced diet, and decreased alcohol consumption, can reduce the risk of developing a chronic disease [2]. Traditional lifestyle interventions have been shown to be effective in helping people adopt a healthy lifestyle [3,4]. However, these interventions mostly focus on the general population and often disregard vulnerable groups, such as those with a low socioeconomic status (SES; people with a low income or low education, or who are from deprived neighborhoods). There is firm evidence that people with a low SES often engage in more risky lifestyle behaviors and have an increased risk for various chronic diseases and premature death than those with a high SES [2,5-7]. Health inequalities for low SES are associated with a reduced life expectancy of 5 to 10 years and a reduced disability-free life expectancy of 10 to 20 years [8]. Furthermore, it seems that low income and poverty are more often associated with poorer mental health [9,10]. A systematic review by Bull et al [11] found that when lifestyle interventions focus on people with a low SES, most result in small and variable effects [11]. These findings may be due to designers not tailoring lifestyle interventions specifically to people with a low SES or not taking into account their specific characteristics and needs [12,13]. For instance, compared to the general population, individuals with a low SES living in poverty may focus more on coping with their current stressful everyday life (ie, money-related stress and unfavorable work environment). These stressors can lead to choosing unhealthy coping strategies, such as tobacco smoking [14], which could be detrimental to long-term health [14]. These challenges call for a different approach to help this priority group adopt and maintain a healthy lifestyle.

eHealth interventions could proactively support people with a low SES to adopt lifestyle changes [15]. The use of the latest information and communication technologies, such as websites, smartphones, email, text messaging, tablets, and smartwatches [16], offers health professionals and researchers more options to tailor intervention content to the specific needs and characteristics of the user [17]. Furthermore, eHealth interventions can provide users with the information, skills, and resources needed for a positive lifestyle change efficiently and interactively [18]. Health practitioners can reach diverse populations more easily with these interventions than with traditional interventions [18]. eHealth interventions can be supported by video or audio and delivered in an accessible manner to patients for use in their own time and home environment [19]. However, current eHealth interventions require users to have good digital skills and a high literacy level, which are often lacking in low SES groups. Moreover, such interventions must consider their different life situations, health care needs, and eHealth expectations [20]. When eHealth interventions do not consider the needs of this target group, intervention uptake can hinder and reinforce the inequitable use of eHealth, exacerbating health inequalities [21,22].

Studies have shown promising results for eHealth among people with a low SES [23]. For instance, Brown et al [23] showed that their eHealth lifestyle intervention for low SES individuals yielded small but significant changes in behavior. Hayba et al [24] suggested that even modestly effective interventions, sustainably deployed to target vulnerable groups (eg, low SES groups), would add value to the field of public health. Even though there is a growing body of research on eHealth lifestyle interventions for this vulnerable group, there is a lack of insight into how eHealth interventions are currently developed, used, and implemented for people with a low SES. Recently, there has been an increased focus on the specific needs and characteristics of low SES groups to bridge this digital divide. For example, the World Health Organization’s digital intervention guidelines for eHealth usage to improve patient care devoted special attention to the needs, preferences, and circumstances of vulnerable groups, such as people with low (digital) literacy skills [25]. However, current guidelines fall short for researchers and developers who want to develop eHealth lifestyle interventions tailored to people with a low SES. Therefore, this scoping review aims to identify intervention components, barriers, and facilitators in the development, reach, use, evaluation, and implementation of existing eHealth lifestyle interventions for low SES populations.

## Methods

### Scoping Review Methodology

We conducted a systematic scoping review from June to September 2019. In July 2021, we updated the search following the same procedures. There were no restrictions on the date of publication for articles retrieved upon searching the databases. Since the research area of eHealth lifestyle interventions for low SES groups is still in its infancy, a scoping review method was chosen because it is an appropriate methodology to map key concepts and identify knowledge gaps. A scoping review also offers the opportunity to review published literature with different methodological designs. It further examines the existing literature concerning the volume, nature, and characteristics of the primary research [26]. We used Arksey and O’Malley’s methodological framework as a guide for the review [26].

### Search Strategy and Eligibility Criteria

We defined the following 5 categories based on 2 frameworks used for the development process of eHealth interventions: development, reach, use, evaluation, and implementation [27,28]. The first framework is the Center for eHealth Research (CeHRes) roadmap, a framework for eHealth development, implementation, and evaluation that combines and uses aspects from approaches like human-centered design, persuasive technology, and business modeling [27]. The second framework is RE-AIM (reach, effectiveness, adoption, implementation, and maintenance), which describes the stages in intervention development and implementation [28]. The categories *development*, *use*, and *evaluation* were derived from the CeHRes roadmap, and the categories *reach* and *implementation* were derived from the RE-AIM framework.

After we defined the scope of the review, we developed a search strategy together with an experienced librarian and domain experts (Multimedia Appendix 1). We searched PubMed, MEDLINE (Ovid), Embase, Web of Science, and the Cochrane Library, using a combination of the following key constructs: eHealth, lifestyle (physical activity, nutrition, alcohol, smoking, and sleep), low SES, and development, reach, use, evaluation, and implementation. The definitions of these key constructs are provided in Multimedia Appendix 2. These databases were chosen because they cover a wide range of scientific articles on eHealth. For each construct, several keywords (spelling variations and synonyms) were used. Exclusion and inclusion criteria were defined based on relevant literature and in consultation with domain experts, after which relevant studies were selected (Textbox 1).

**Inclusion and exclusion criteria for selecting the studies.**

**Inclusion criteria**

- Description of an eHealth/web-based intervention or mHealth/telemedicine intervention
- Definition of socioeconomic status (SES) as the position of an individual on a socioeconomic scale that measures factors by a single variable, such as education, income, or neighborhood status, or multiple variables
- Focus on at least one lifestyle component (physical activity, diet, alcohol, smoking, sleep, or overweight)
- Targeting of a low SES population (>18 years of age)
- Presentation of information on development, use, reach, evaluation, or implementation
- Publication of full text in English
- Any study type (included study protocols)

**Exclusion criteria**

- Measurement of SES using other variables (eg, race and ethnicity)
- Conference abstracts and reviews presenting filtered information, such as systematic reviews, scoping reviews, and narrative reviews

### Data Extraction and Analysis

The eligibility criteria were used to review the articles. Initially, IA screened the titles and abstracts for the first selection of articles. Then, IA checked the bibliographic reference lists of publications that remained after full-text selection to identify any additional eligible publications. Any doubt about the included studies was discussed with the other authors. We extracted general study characteristics (eg, the year of publication and country), and details on SES, effectiveness, development, reach, use, evaluation, and implementation. Data were extracted as barriers or facilitators if they were related to the development, reach, use, evaluation, or implementation phases of the intervention and they were identified or mentioned as facilitators or barriers by the included studies. Even if the barriers and facilitators were mentioned in one of the included studies, they were eligible for inclusion. If there were uncertainties concerning under which phase the barriers and facilitators fell, they were discussed with the other authors.

Furthermore, we selected additional categories based on the CONSORT-EHEALTH checklist (V1.6), which provides helpful guidance on what eHealth studies should report [29]. These added categories were the (behavioral) theories or models used to develop and evaluate an eHealth intervention. The categories also included the level of human involvement in the intervention (eg, automated or human guidance) during the development, evaluation, and implementation (eg, health professionals and researchers).

The selected articles were mapped, and data were recorded in Microsoft Excel (Multimedia Appendix 3). Data were synthesized narratively, and the findings were then summarized and grouped into themes as defined by the authors.

## Results

### Study Selection

**Figure 1:**
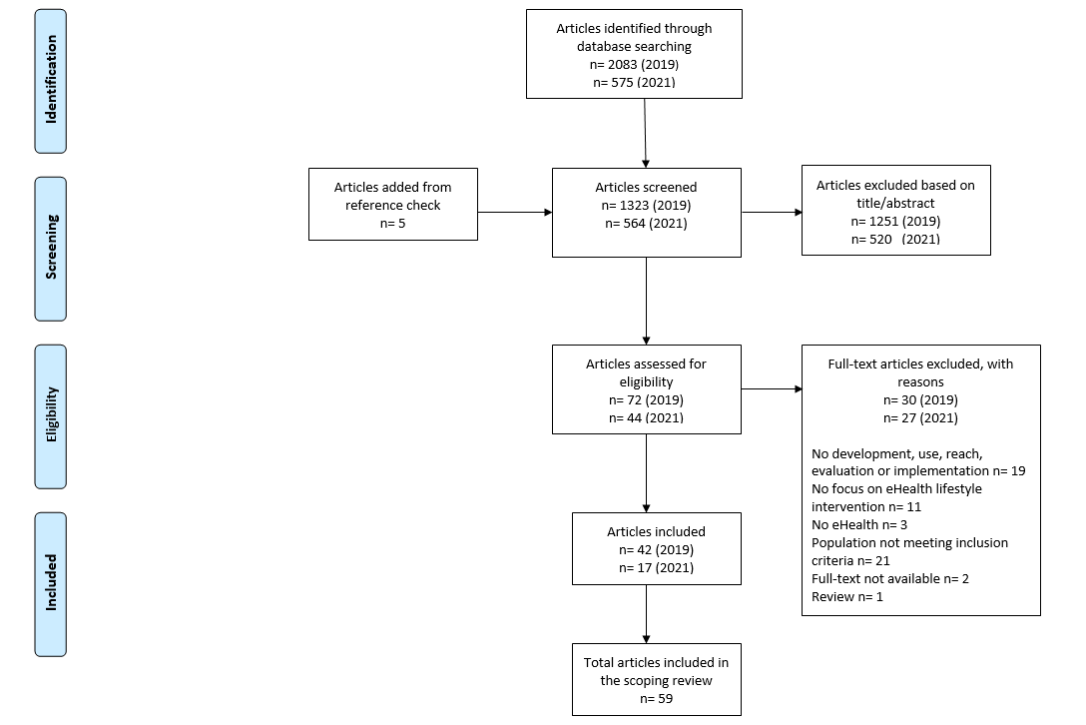
Flow diagram of the article selection process.

The systematic search across the databases revealed 2083 potentially relevant citations. After removing duplicates (n=765) and screening 1323 titles and abstracts, 72 full-text articles were screened for eligibility. Of these, 42 articles met the eligibility criteria and were included in this review (Figure 1). The updated search led to 17 articles that were included in this review.

### Description of the Included Studies

The studies included were randomized controlled trials (RCTs) [23,30-40], observational studies [41-56], and design studies [57-77]. Several RCTs and observational studies evaluated eHealth interventions on health outcomes (eg, BMI, blood pressure, and hemoglobin A_1c_) [30, 34, 35-37, 40, 43, 45, 47, 48, 53, 78], nutrition-related behavior outcomes [32, 38, 39, 79, 80], physical activity–related outcomes, smoking-related outcomes [23,31,46,51,81,82], usage outcomes [33,49,54], and reach [44], as well as feasibility and acceptability outcomes [47,80] (Multimedia Appendix 3). Design studies examined recruitment [57], usability, feasibility [57-65], development, or acceptability of eHealth interventions [58-60,64,65,67,68,70]. The interventions were aimed at weight loss (n=9), physical activity (n=9), healthy eating (n=11), smoking (n=13), and alcohol use (n=2), and 17 interventions focused on multiple behaviors. The target audience of these interventions was mainly low SES participants; several studies also targeted a highly educated population [31-33,36,44,53,82].

The different studies assessed the education level [23,30-33,36,40,44,46,53-55,78,82], occupation [23,67], or income level of the participants [43,45,48,51,73,74]. In addition, the participants were recruited from a low SES neighborhood (residents who were unemployed, had a low education, or had a financial disadvantage) [60,64]. A summary of the study characteristics is presented in Table 1.

**Table 1:** Summary of the study characteristics (N=59).

First author, year	Study design	Target population	Type of eHealth technology
Aguilera [83], 2020	Protocol	Low income^a^	App (and SMS text messaging)
Aldoory [71], 2016	Design	Low income	SMS text messaging
Athavale [72], 2016	Design (part of RCT^b^)	Low income^a^	Web-based
Atkinson [66], 2009	Design	Low income	Web-based
Bond [65], 2021	Design	Low income^a^	Web-based and SMS text messaging
Brown [23], 2014	RCT	Low SES^c^	Web-based
Griffin [48], 2020	Observational	Low income	SMS text messaging
Brunette [81], 2015	Quasiexperimental	Socioeconomically disadvantaged^d^	Web-based (single session)
Burner [80], 2020	Quasiexperimental	Low income^a^	SMS text messaging
Carolan-Olah [64], 2021	Design	Low SES neighborhoods	Web-based
Cavallo [47], 2021	Observational	Low income	Social media
Lepore [49], 2021	Observational (part of RCT)	Low income^a^	App
Stanczyk [31], 2013	RCT	Low, middle, and high education	Web-based
Clarke [39], 2019	RCT	Low income	App
van Dijk [84], 2021	Protocol	Low SES^a^	Web-based on smartphone
Brown [46], 2012	Observational	Low SES^c^	Web-based
Evans [62], 2019	Design	Low income	App
Flaherty [67], 2020	Design	Low SES^e^	App
Delrahim-Howlett [38], 2011	RCT	Low income	Web-based
Fontil [61], 2016	Design	Low income	Web-based
Garvin [73], 2019	Design	Low income	App
Golsteijn [85], 2017	Protocol	Low, middle, and high education	Web-based
Foley [37], 2016	RCT	Socioeconomically disadvantaged	SMS text messaging
Greene [54], 2021	Observational (secondary data analysis)	Low and middle educated	App
Cavallo [45], 2016	Observational	Low income	Web-based and social media
Tagai [50], 2020	Observational	Low income	SMS text messaging
Golsteijn [36], 2017	RCT	Low, middle, and high education	Web-based
Griffin [43], 2018	Observational	Low income	SMS text messaging
Kim [86], 2018	Nonrandomized design	Low income^a^	Web-based
Kothari [68], 2020	Design	Low income^a^	App
Leak [63], 2014	Design	Low income^a^	Social media
Kendzor [51], 2020	Observational	Low income	App
van Dongen [44], 2012	Observational	Low, middle, and high education	Web-based (email)
Lohse [52], 2013	Observational	Low income	Social media
Mayberry [74], 2016	Design	Low income	SMS text messaging
Michie [55], 2012	Design and observational	Low SES	Web-based
Neuenschwander [79], 2013	Block equivalence randomized trial	Low income	Web-based
Pathak [75], 2021	Design	Low income^a^	App (and SMS text messaging)
Patten [87], 2019	Nonrandomized design	Low income	SMS text messaging
Radhakrishnan [53], 2016	Design and observational	Low, middle, and high education	App (mobile device video game)
Herring [35], 2014	RCT	Low income^a^	Web-based (social media) and mobile phone (text messaging)
Régnier [60], 2018	Design	Low SES neighborhoods	App
Ramirez [34], 2017	Pilot RCT	Low income^a^	Text messaging or voice
Silfee [56], 2018	Design and observational	Low income	Web-based
Silfee [42], 2019	Observational	Low income^a^	Web-based
Silk [41], 2008	Observational	Low income	Web-based vs games
Simons [58], 2018	Design	Lower education	App
Simons [40], 2018	RCT	Lower education	App
Spears [59], 2019	Design	Low SES	SMS text messaging
Schneider [33], 2012	RCT	Low, middle, and high education	Web-based
Stanczyk [82], 2014	Data from RCT	Low, middle, and high education	Web-based
Springvloet [32], 2015	RCT	Low, middle, and high education	Web-based
Stotz [76], 2018	Design	Low income	Web-based on smartphone
Tabak [77], 2018	Design	Low income^a^	SMS text messaging
Lohse [57], 2013	Design	Low income^a^	Social media
Wayne [30], 2015	RCT	Low SES	App
Wayne [78], 2014	Single arm	Low SES	App
Whittemore [70], 2020	Design	Low income	SMS text and MMS messaging
Yee [69], 2020	Design	Low income^a^	SMS text messaging

^a^Socioeconomic status was not specified in the study.

^b^RCT: randomized controlled trial.

^c^SES: socioeconomic status.

^d^Low education, unemployment, or living in poverty.

^e^Socioeconomic status was determined by the occupation and employment status of the household’s primary income earner.

### Intervention Development and Evaluation

In the various stages of the development and evaluation of the intervention (ie, problem definition, development, and implementation for the study) [48,50,59,60,64,66,69,70,84], several studies involved stakeholders, which included family members, experts, key informants [50,61,69,75,84,86], health professionals, and end users [48,54,64-66,68-70,73,75,83]. However, some studies provided little information on the identification of stakeholders and did not clarify the level of involvement of stakeholders and end users [37,44,50,56,66,70,78,79,81,83,87]. The studies used multiple methods, such as interviews, focus groups, and user testing [50,54,57,64-70,73,75,83], to gain insights from end users and stakeholders. Researchers used focus groups to map the needs and problems of the (potential) users [58,59,61,62,66,68,70,73] and to gain input from stakeholders to adapt existing interventions [61,70,77,86]. These methods also helped the researchers to gain insight into the challenges that participants experienced while using the intervention [65,73,86] and their thoughts on the requirements of successful participation [65,66,68,77,86]. Furthermore, other methods used the Community Engagement Studio [74], a consumer panel [32], and a collective discussion group [60]. The researchers used these methods to improve the accessibility of the interventions for the end users [60,74]. For the development phase, facilitators and barriers were related to technology and content factors. However, regarding the evaluation of the interventions, limited facilitators and barriers were mentioned. Several studies adapted existing interventions, which were developed and tested in different SES groups with various health concerns, such as diabetes, hypertension, mental disorders, and pregnancy [38,45,47,49,56,57,61,70,72,77,79,81,85-87].

Studies adapted these interventions and the delivery modality for use in different low SES groups. Nevertheless, many studies retained most of the content and components of the existing interventions [45,56,57,61,70,72,77,79,81,87]. Many studies chose to adapt the content of the intervention and apply linguistic and content simplification, such as using plain language and low content load through the use of images and videos [57,61,70,81,86,87]. Some studies also made cultural adaptations by using updated cultural components [61,86], translating the content into a second language (eg, Spanish), and employing bilingual coaches [61,86]. Although intervention adaptation was common, documentation of the adjustment process was scarce. Only 3 studies [70,77,85] described in detail the adaptation process and what changes they performed. Furthermore, 2 studies used frameworks (Stirman and the intervention mapping protocol) [77,85], and 1 study [87] used a model (Stage Model) to adapt the intervention.

### Tailoring

The majority of the studies tailored the eHealth interventions in various ways to the characteristics and skills of people with a low SES [23, 31-37, 40, 45, 46, 50, 58, 61, 62, 64, 66, 69-72, 74-77, 81-86, 88]. One method of customizing the eHealth intervention matched the content delivery (eg, visual or text information) to the user’s language and digital literacy skills [34,50,54,61,64,66,69,70,75,80,84,86]. Another method tailored feedback, advice, and information to the characteristics (eg, cultural adaptations and practical advice relevant to their situation) of individuals with a low SES [23,31,33,35-37,39,40,45-47,58,64,66,74,75,77,81-83,85] or the timing and type of text messages (eg, feedback) [83]. However, it is unknown how tailoring was applied (technology or human tailoring, or a combination of both methods). A few studies based tailoring on theoretical models of behavioral change [31-33,46,70] and gathered information through questionnaires [31-33,36,40,47,55,58,62,66,85], self-monitoring data [23,37,46,55,77,83], or intervention goals [32,74]. However, tailoring the intervention system to deliver feedback or advice proved challenging as it required technological expertise and financial recourses [33,40]. It is unclear whether tailoring led to better results. Because of tailoring the feedback, 1 study showed that lower-educated smokers were more likely to revisit the intervention website [82].

### Reach

The included studies applied multiple strategies to recruit low SES participants. However, some studies (n=5; 8%) provided limited details on the strategies they used to reach their participants [35,38,39,62,79]. The recruitment strategies and places are summarized in Table 2.

Different methods were found to be helpful to reach low SES participants. Lohse et al [52] found that Facebook is an effective tool to reach low-income women. Furthermore, the studies that used a personal approach to recruit participants reported a higher enrollment rate [61,82,86,87]. For example, Patten et al [87] found that reaching the targeted population with a face-to-face outreach method was more successful compared to recruitment through flyers. Kim et al [86] found that personal or telephone approaches to recruiting participants were responsible for most of the enrollments in their study. Moreover, participants indicated that they were more receptive to participating in a study when their doctor had previously discussed it with them [86]. Another study found that smokers recruited through general practitioners were more likely to be lower educated and already living with smoking-related illnesses than participants recruited through the internet [82].

Some studies experienced challenges in reaching low SES groups. These studies reached mainly medium or highly educated [33,44] participants with stable incomes and relatively healthy lifestyles [33]. van Dongen et al [44] indicated that people with a low SES may be reached with the right strategies, such as integrating an eHealth intervention into standard midwifery care, increasing awareness about the intervention’s existence by expanding mass media use, and involving key community representatives of the target group. Additionally, some studies recommended increasing the reach of lifestyle interventions by collaborating with other experts, such as designers and health professionals [44,61,62]. Tables 3 and 4 show the barriers and facilitators for reach found in the studies.

**Table 2:** Participant recruitment, places, and strategies.

Recruitment characteristic			Number of studies	
**Individuals involved in recruitment**				
	Health professionals [30,34,42,44,74,78,81-85]^.^	11		
	Researchers [37,40,58,61,81] and research assistants [38,40,80]	8		
	Study coordinators [86], managers [40,41], organization staff [68], and agent assistance [48]	5		
	Paraprofessionals [63,79] and volunteers [60,66]	4		
	Snowballing (*participants recruited other participants*) [58,65,67,82]	4		
**Recruitment places**				
	Health care setting [30,35,44,45,53,54,59,61,64,65,68,75,78,80,81,83-86]	19		
	Federal Benefit and Assistance Program for low-income women [38,42,49,50,56,72,73] and families [43,47,48,57,79]	12		
	Workplaces [40,77] and care services [34,60]	4		
	Local communities [59,66,68,71,87]	5		
	Food bank distributors [39,43,62]	3		
	Public health insurance [70]	1		
	Local nongovernmental organizations [51,60]	2		
	Public places [47,67]	2		
	Research agencies [31,71]	2		
**Recruitment strategies**				
	Online [23,30,32,37,40,43,44,46,47,51,52,55,58,59,61,65,68,73,74,76-78,82,84-87]	27		
	Newspaper advertisements [33,85], banners [44], flyers, and posters [45,47,50,57,59,61,66,68,77,78,83,85,87]	16		
	Personal contact (face-to-face) [33,40,45,58,65,67,68,74,80,83] or via phone [51,66]	12		
	Postal invitation letters [32,37,57]	3		
	Local television campaigns [32,82]	2		
	Regional health authority [33]	1		

**Table 3:** Overview of facilitators identified in the eHealth interventions.

Facilitators per phase			Studies
**Development**			
	Iterative design of the intervention (user-centered approach)	[40,46,58,59,61,62,65,66,68,69,74-77,85]	
	Study staff collaborating with other experts or a digital health company	[53,54,61,62,65,69,88]	
	Broad number of data sources to inform development	[65,75]	
	Participants’ knowledge of technology	[60,65]	
	Providing devices	[39,51]	
	Concise and clear content	[54,56-59,61,63,64,66,69,70,75]	
	Use of visual and multimedia elements	[23,30,40,47,56,59,60,63-66,69,70,73]	
	Resonating content of the intervention with participants	[53,60,61,66,85]	
**Evaluation**			
	Conducting formative evaluation in the early stage of the intervention	[40,41,46,55,58,61,62,65,66,68,70,74-76,85]	
**Reach**			
	Recruitment through Facebook, and active recruitment through health care professionals and tailored recruitment strategies	[44,47,52,65,82,87]	
	Collaborating with other experts, such as designers and health professionals, and local community services	[44,61,62,66,75,76]	
**Use**			
	Social support (friends, family, and peers)	[34,39,45,50,56,59,60,62,63,69,74]	
	Self-monitoring	[34,45,48,49,58,61,66,67]	
	Human coach can be helpful for participants	[30,47,49,50,56,57,87]	
	Practical advice to incorporate a healthy lifestyle in daily life	[54,56,58,60,63,64,66,73]	
	Reminders	[33,45,53,59,67,69]	
	Trust (eg, have a familiar face posting on a social media page) and credible information	[54,60,63,66,69]	
	Recipes and meal ideas may be useful	[47,54,66,69,73]	
	Helping participants with technology use	[57,61,66,86]	
	User friendliness and simplicity	[64,66,70,73]	
	Interactive features	[64-66,68,69]	
	Providing incentives and rewards (eg, virtual or financial rewards)	[35,48,53,58]	
	Links to more information	[64,66,69]	
	Combining social media with face-to-face group sessions	[47,56]	
	Networking with others encourages participants’ use of social media interventions	[56,63]	
	Activities must focus on pleasure and not obligation	[61,66]	
	Incorporating affordable options	[61]	
**Implementation**			
	Supplying the intervention through different platforms	[36]	
	Increasing direct communication with the health coach	[61]	
	Training health care professionals	[70]	
	Collaborating with health insurance	[70]	
	Server support staff and marketing team continually monitoring the intervention for technical issues	[76]	

**Table 4:** Overview of barriers identified in the eHealth interventions.

Barriers per phase			Studies
**Development**			
	Technical challenges with the intervention software or prototype	[53,58,60,62,70,71,78,86]	
	Amount of information or visuals	[40,56,59,63,66,68,85]	
	Limited financial resources for the intervention	[39,53,58,72]	
	Optimal frequency for reminders or messages	[33,59,68]	
**Evaluation**			
	Evaluation is time-consuming	[62]	
	Slow iterations of the intervention in the academic field	[65]	
**Reach**			
	Introductory study presentations and sending reminders to clinicians had a limited effect on increasing referrals	[86]	
	Passive recruitment (flyers)	[87]	
**Use**			
	Technical difficulties using a self-monitoring device or eHealth intervention (eg, lack of internet access, problems with telephones, and poor signal)	[34,40,45,49-51,56,58,60,61,65,66,68,70,71,73,85]	
	Limited digital skills of users and lack of knowledge of innovative technologies	[34,60,61,65,66,86]	
	Not wanting extra push notifications and lost notifications among all the notifications from other apps	[40,58,68]	
	Not allowed to carry a smartphone during work or does not carry a phone	[58,75]	
	Literacy and not mastering the language	[32,34,60,66,70]	
	Lack of time in a low SES^a^ group	[34,45,49,54,56,60,61,68,69,73,78]	
	Financial problems (eg, paying bills)	[60,68,69]	
	Lack of familiarity with other participants before using social media and trust in social media or the internet	[45,60]	
	Waning participant interest toward the end of the intervention period and low motivation	[40,49,61]	
**Implementation**			
	Limited time of staff or coaches	[39,53,74]	
	Limited financial resources	[39,72]	
	Difficulties getting medical data of participants from participating health care facilities	[86]	
	Limited ability of peer coaches	[72]	

^a^SES: socioeconomic status.

### Use of eHealth Interventions

Most studies did not mention how the participants used the eHealth lifestyle interventions. However, many studies gained insight into the intervention usage by evaluating the concepts of adherence, user engagement, and acceptance [23,40,46,47,49,53,54,56,58,59,61,71,72,74,80,81]. Most of the studies showed that participants with a low SES accepted the eHealth interventions [40,46,47,53,54,56,58,71,74,80]. When there was high adherence, usage, and user engagement, interventions seemed effective [23,81].

Several studies mentioned explicitly measuring intervention usage with Google Analytics (eg, user interactions with content) [40], log data [23,47,54,61], registration data [44], emails sent, quiz questions accessed [44], questionnaires [39,40,44,64], or self-monitoring questionnaires [39]. The data analysis demonstrated that interventions were used as intended [23,61,81]. However, Régnier et al [60] found that the intervention was used to a less extent due to different barriers, such as technical issues, lack of language skills, and searching for real contact. In addition, Simons et al [40] reported decreased use during the intervention because of lesser engagement with the intervention. It also emerged that there was a difference between users within the interventions [33,40,81]. For example, in a study, it was found that the users who received notifications with tips, facts, and feedback mostly used the intervention [40]. Using periodic email prompts significantly increased the reuse of the intervention [33]. Schneider et al [33] concluded that it is crucial to develop strategies that encourage engagement from people with a low SES. Furthermore, hedonic elements (eg, visual elements) in the intervention were significantly associated with increased use [54]. In another study, personal and nuisance factors were associated with lower intervention use, including lower educational achievement and perceived barriers (eg, no time or interest and technical problems) [49]. Barriers and facilitators for using the interventions were diverse and varied in terms of individual and technological factors (Tables 3 and 4).

Other studies have analyzed adherence to interventions [31,35,49,87] by measuring the numbers of messages sent by participants, completion of coach calls [35,87], or intention to visit or revisit the intervention, or using specific features of the intervention, self-monitoring data, and days that participants used the intervention [31,49]. Adherence to the intervention decreased gradually in certain studies [35,49]. Griffin et al [48] showed that noncompleters of the intervention had certain characteristics (were younger, were African American, had a high BMI, had a lower education [high school or lower], and had a low income) when compared with participants who completed the intervention. Engagement with interventions was measured through the self-management behavior of participants [86], the tracking of their behavior via self-monitoring devices [86], self-reporting [56,64], and the presence of several likes, comments, and posts or messages assessed throughout the intervention delivery [47,56,59,72,86], as well as by capturing the frequency of user logins [86]. At the time when intervention engagement was high in several studies [59,61,72], in other studies, engagement decreased during the use of the intervention [45,47,51,71]. In 1 study, participants were more engaged with text messages than voice messages [34]. Another study showed that participant contributions appeared to vary across time of the day and day of the week (more active in the beginning part of the week and during the middle of the day) [47].

### Delivery Mode of the Intervention

There was a wide variation in the delivery mode of the intervention. Table 1 provides an overview of the modes used to deliver the intervention. The studies cited several reasons for using a certain delivery mode. Using the internet [23,31,33,42,44,45,55,56,61,63,66,79,81,86], smartphone apps [58,60,73,75,78], or text messages [34,35,50,70,71,75] offers many benefits. Internet-based [36,42,44,45,52,56,64,86] and text-based [34,43,71,77,87] interventions are good channels for reaching hard-to-reach groups and might be effective in changing healthy behavior [32,34,44,47,48,50,54,59,66,70]. They also help to investigate new channels or to deliver interventions to low SES individuals [33,45,47,53,56,57,74,79]. Finally, low SES individuals use the internet, which provides the interventions an opportunity to reach this target group [39,42,45,52,56-58,62,73,86].

Studies reporting on the effectiveness of the delivery mode demonstrated no unequivocal results. Three studies showed that a web-based intervention was more effective for low SES participants [35,41,79] than non-eHealth interventions, such as in-person counselling for low SES participants [79] and game-based versions [41], and website users had deeper knowledge and a higher intention to use the website henceforth [41]. Another study [53] demonstrated that a gamified intervention significantly improved heart failure self-management knowledge in low SES and high SES participants. Participants with lower education levels and older adults preferred a digital game to any other medium for receiving information on self-management of heart failure. One RCT reported no interaction effects between delivery strategy (video versus text advice) and education level in terms of message processing mechanisms and future use of a smoking cessation intervention [82].

### Implementation

Most studies did not adequately describe how their respective eHealth lifestyle interventions were implemented, perhaps because almost all the interventions were pilot projects and were not implemented in practice after the study was completed. The few barriers and facilitators that were identified are listed in Tables 3 and 4.

Several studies reported that they collaborated with different disciplines for implementation [55,61,70,72,77,85]. Golsteijn et al [85] created a network of hospitals and radiotherapy institutes to implement the intervention. However, their results on implementation are unknown. Furthermore, it appears that health professionals play an important role in the implementation of interventions [70].

Very few studies discussed the cost of their eHealth interventions. Limited finances and staff time presented several challenges in implementing these interventions [39,72]. Tabak et al [77] considered practicality and sustainability of the intervention by choosing interventions that prevented higher cost, for example, providing automatic feedback instead of individualized feedback. Other examples include finding enough coaches with the expertise to guide participants [72] and working with their time constraints [74]. Studies that reported on how they evaluated the implementation of their interventions were scarce. However, 1 study [85] used intervention mapping to develop an implementation plan. Two studies plan to evaluate implementation in the future. Whittemore et al [70] aimed to document an implementation analysis, and Foley et al [37] aimed to evaluate implementation through the RE-AIM framework.

### Effectiveness

A number of studies (n=19) [23, 30, 32, 33, 34, 35, 36, 38, 39, 41, 43, 45, 46, 48, 50, 79-82] investigated the effectiveness of interventions for smoking cessation [23,46,51,81], healthy eating behaviors [32,39,79], alcohol [38,80], weight loss [35,37,45,47], physical activity [34,36,40], and multiple lifestyle changes [30,33,43,48,78]. Three studies [23,46,81] were effective in achieving smoking cessation in the low SES group. Furthermore, some studies reported significant improvements in eating behaviors [32,39,43,79], reduction in weight [35,43,48], and increase in physical activity [34,36,48]. Two RCTs showed that interventions were more effective in high SES participants than in low SES participants [32,36]. One study [32] found educational differences in high-energy snack intake. In this previous study, the plus group (environmental-level factors) received information on the availability and location of healthy food in the home environment and the prices of healthy food products in the supermarkets that the participants usually shop at. The plus approach targeted higher-educated participants more effectively than the basic approach, which was more effective for lower-educated participants. The authors argued that higher-educated participants understood and applied the environmental-level information easier than the lower-educated participants. The intervention as described by Golsteijn et al [36] resulted in a significant improvement in self-reported physical activity. However, the highly educated group initially participated more on the web than their lower-educated peers. In contrast to a study, they found minor effects in low SES participants, but no effect in participants with a higher SES [23]. The authors stated that this is likely because the user testing of the intervention was conducted exclusively with smokers with a low SES, which contributed to its effectiveness in the low SES group.

Two studies [33,45] reported minor significant improvements and modest effects on reuse of a healthy lifestyle program [33]. Other studies reported an insignificant effect [40] due to lower user engagement and dropouts.

### Intervention Components

Studies applied diverse components within the interventions. For example, they employed visual and multimedia elements, such as images, infographics, videos, and social support. To a lesser extent, there was human or virtual coaching, and incentives were used. Table 5 presents an overview of the components in eHealth lifestyle interventions.

**Table 5:** Overview of the eHealth lifestyle intervention components (N=59).

Components	Studies, n (%)
Multimedia (images, infographs, and videos) [23,30,31,35-38,41,46,47,49,55-57,59-66,68,70,76,78,79,84-86]	30 (51)
Self-monitoring [23,30,34-37,40,43,45-49,51,56,58,60,61,65,67,70,76-78,83,84,85,86]	28 (47)
Tips [23,38-40,43-45,48,49,53,54,58,60-62,64,66,68,71,75-77,79,84-88]	28 (47)
Social support [33,34,36,37,40,43,45,47,49,53,55,58,61,62,67,69,71-74,77,84-87]	25 (42)
Reminders [23,30,32,33,36,37,40,45,46,48,49,51,53,55,58,59,65,67,69,78,84-86]	23 (39)
Rewards/incentives [32,34,35,38,41,45-48,50,51,53,56,64,68,73,74,76,79,80,83,87]	22 (37)
Coach [30,35,37,47,51,56,61,66,70,72,74,75,77,78,84-87]	18 (31)
Theoretical frameworks [23,31-34,37,43,46,48-50,55,64,69,70,73-76,80,81,83,85]	23 (39)

#### Theoretical Frameworks

Several studies (n=23, 39%) stated that they used one or more theoretical frameworks in their interventions [23,31-34,37,43,46,48-50,55,64,69,70,73-76,80,81,83,85]. The frameworks most commonly used were the social cognitive theory [34,37,43,48,64,70,73,85], I-Change Model [31,33,85], and theory of planned behavior [32,81,85], followed by the Health Belief Model [69,76,85], theories of self-regulation [32,85], and Precaution Adoption Process Model [32,85]. However, several studies mentioned using the Techniques of Behavior Change [23,45,46,67,77]; the theories for the rest of the studies can be found in Multimedia Appendix 3. Few studies used the frameworks to develop, adapt, evaluate, or implement the eHealth interventions [37,56,76,79]. It is unclear whether these theories were associated with desirable effects. Although not all studies have reported why they chose the theories [33,53,82], a few mentioned using the constructs or determinants of the theories [32,37,43,81,85], due to their suitability and available evidence [23,34,40,46,55,58,85]. Furthermore, it appeared that some interventions included components, such as self-monitoring, reminders, and social support based on behavioral strategies or theoretical frameworks, to promote lifestyle change or maintain healthy behaviors.

#### Multimedia and Visual Elements

Many studies included multimedia in their interventions, such as videos [35, 36, 37, 61, 79, 85, 86] or images [23, 38, 40, 46, 53, 55, 59, 62, 63, 89]. Although it was unclear why studies included these materials; some used videos [35-37,61,79,85,86] to introduce the intervention components [37,61], provide skills training [37,79], give home exercise instructions [36,85], or introduce the participants to their coach [86]. Other studies used visual materials, such as images and videos, to increase engagement [56,76]. Interventions applied images because of their visual appeal and ease to recall [55], or to enhance learning and motivate users to continue using the program [76]. There was almost no mention of using graphic artists [62,76] or photographers [76] to create illustrations for the interventions. However, Evans et al [62] stated that selecting illustrations for the app was challenging because matching the main text with illustrations was not always easy and required more iterations to meet the criteria. It was also challenging to find the right graphic artist to design proper images based on the given assignment. In 1 study [56], long videos resulted in lower engagement with Facebook participants. Another qualitative study [60] found that participants who experience language barriers rely more on visual materials (ie, videos) than written materials. However, a study reported that illustrations crowded with visual details confused participants [62]. Another study [63] highlighted that participants emphasized the importance of photos and visual appeal. In the study by Silfee et al [56], participants were more likely to read and comment on Facebook posts containing messages with images. One study [40] made it possible for the participants to see their daily steps via graphs. Although participants appreciated graphs, they used them significantly less at the end of the intervention due to decreased interest and outdated graph data. Only 1 study chose audio to increase the media on the website and facilitate relapse prevention and coping [55].

#### Social Support

Participants’ peers [35,45,47,56,61,63,86] or significant others [34,72,74,87] provided social support, online or offline [35,45,56,61,63,71,86]. Other studies only gave advice on how people can get social support to help each other to adopt new behaviors [40,59,70,85,88]. Three studies mentioned that participants had positive experiences with the social support provided by their significant others [34] or peers [47,56,71] (they perceived a sense of community and social [71], emotional, and instrumental support [34]) and that peers motivated them [56]. For others, the ability to network and interact with peers was an important reason to visit the social media of the intervention [63]. However, it is difficult to determine whether social support contributed to the increased effectiveness of some interventions.

Several studies provided support through social media [35,45,56,61,63,86]. Participants were part of an online social network where they could, for example, discuss their goals [61,86] and challenges [86], and offer each other social support [35,47,61]. However, the studies identified different challenges in supporting active participation in the social support component, such as lack of a connection with other participants before accessing the eHealth intervention, limited engagement with other participants on social media [45,61], and not receiving timely responses from other participants [63]. Furthermore, in a qualitative study [61], participants experienced their level of literacy as an obstacle to taking part in online discussions, while in another study, posting about themselves made some participants with low SES uncomfortable [56], and others did not want to share their unsuccessful weight loss [56]. Involving support persons in the intervention appears to be complicated; some participants with low SES had no support person or did not want to involve one [34,74]. Furthermore, Pathak et al [75] showed that participants who had no family disliked messages that alluded to family support, and the term was replaced by loved ones (similar to familial relations). The interventions [45,56,63] offered many solutions to encourage the use of social support on social media, such as team-building exercises and enlisting friends [45]. The majority of participants of a smoking intervention relapsed, nonrelapsers reported significantly less temptation to smoke, and the qualitative data showed that nonrelapsers were able to manage temptation and reported greater support [50].

#### Self-monitoring

Several studies used few self-monitoring devices, based on emerging evidence or previous studies [37,85], such as pedometers [34,43,45,56,61,85,86] and weight scales [37,43,45,56,61,86]. Participants with a low SES monitored their diets digitally [30,60], with a calorie-counting book [45], or kept paper records [34,56]. Physical activity was also tracked through Fitbit devices [40,58,77] and MyFitnessPal [56]. Participants entered self-monitoring data [30,43], or this was done automatically [30,37,40,56,77,86]. Simons et al [40] found that continuous engagement with a self-monitoring device was challenging, due to participants not wearing the tracker or forgetting to charge it. Few studies provided information about the participants’ experiences, or why the studies chose self-monitoring devices. However, some studies mentioned that participants found self-monitoring devices easy [40,61] and comfortable to use [40,58]. In 2 studies, participants struggled to use tracking devices [34,56], while in another study, participants desired digital apps for calorie counting [45]. It is difficult to determine whether self-monitoring led to increased effectiveness of the intervention. However, 1 study found that food photo journaling improved dietary choices more than having a health coach only [30].

#### Reminders

Sending reminders to participants was used by many studies; however, it is unclear in some studies how they applied the reminders in their interventions [32,45,53,71]. Two studies applied reminders to improve the adoption of and adherence to healthy behaviors [30,78] and to improve heart failure self-management skills [53]. Other studies applied reminders to encourage participants with low and high SES to visit or revisit the intervention [32,33,86], to remind users about their goals [58,85], and to remind users to submit their self-monitoring information [37]. Reminders were often used in the form of automatic emails [33,46,86,89], push messages via smartphones [40,58,78,86], text messages via mobile phones [37,59], and news updates [85]. The majority of studies did not report on how the participants evaluated the reminders. However, 2 studies showed that participants with a low SES found reminders helpful [53,58]. Furthermore, 2 studies indicated that participants had a greater need for reminders [45,59]. Some interventions that employed reminders appeared to be effective [32,33]. For example, in an RCT, reminders increased revisits to the intervention [33].

#### Coaches

Several studies included a coaching component in the intervention [30,35,37,47,51,56,61,66,70,72,74,75,77,78,84-87]. The coaches provided guidance mainly by telephone [30,35,37,61,72,74,75,77,78,84,87], followed by face-to-face counselling [30,37,61,74,78,87], text messages, email [30,61,66,78], online counselling [36,49,56,85,86], or combinations of these methods [30,37,61,78]. This was done through health professionals [30,36,37,72,85], researchers [51,56,74,75,77,87], parahealth professionals [49,72], and automatic phone [37,72]. The roles of the coaches varied and included guiding participants in setting goals [35,37], helping to solve problems [85], and providing behavioral skills training [37,56], and they also stimulated discussions on the online platforms of the interventions [56,86]. Interactions with the coaches varied from single, daily, or regular monthly contact [30,35,37,56,72,77,87] to ad hoc, based on needs [85].

Some coaches were experienced in behavioral change methods [30,35,37,56,76,78,87], and 3 coaches applied motivational interviewing [37,72,87]. It is difficult to determine whether coaching led to increased effectiveness of the intervention. However in 3 studies, the coaching component seemed promising [47,49,56]. The coaching component was positively associated with intervention usage [49] or higher engagement [47]. Furthermore, several studies reported that participants with a low SES appreciated the coaches [56,61,74,87]. Moreover, in 1 study, after the coach stopped engaging on social media (eg, posting and commenting), intervention engagement considerably decreased and passive engagement increased [56].

#### Incentives

Many studies [23, 32, 34, 35, 38, 41, 45, 52, 53, 56, 71, 76, 79, 87] offered participants incentives (eg, gift cards) [23, 32, 34, 35, 38, 41, 45, 52, 53, 56, 71, 76, 79, 87] for completing the assessments [32,34,38,41,45,52,56,71,79,87] to improve response rates [23,52], when submitting their saliva [35,87] or sending their self-monitoring data [35]. Nonetheless, it is unclear whether incentives delivered positive results. In fact, Radhakrishnan et al [53] found that the rewards and incentives offered in a game intervention did not match the real-time behavior, while in another study, participants suggested a greater frequency of incentives [45].

#### Tips

Providing practical information as tips was mentioned in several studies [23, 38-40, 43-45, 48, 49, 53, 54, 58, 60-62, 64, 66, 68, 71, 75-77, 79, 84-88]. Various studies chose this practical component based on theories [55,68,69,75]. Participants appreciated tips or found it useful to receive practical solutions as tips [54,56,58,66,68]. However, tips have to fit into the socioeconomic and sociocultural realities of people with a low SES [60,61,68]. It is unclear whether tips led to increased effectiveness of the intervention. However, Greene et al [54] found that intervention use was significantly higher among those who found the “Tip of the Day” motivating.

## Discussion

### Principal Findings

This scoping review provides an overview of the most commonly applied components in eHealth lifestyle interventions (development, reach, use, evaluation, and implementation) for people with a low SES. It also investigates the most common barriers and facilitators for current eHealth lifestyle interventions. The components that emerged can be classified into behavioral components (such as basic theoretical foundation, coaching, social support, reminders, self-monitoring, and incentives) and technological components (such as visual multimedia, reminders, and self-monitoring). Nevertheless, we found considerable heterogeneity in components, barriers, and facilitators, showing significant variation between studies. Moreover, we believe that the majority of barriers and facilitators for development and use are related to technology (eg, technical difficulties) and environmental factors (eg, financial resources of the intervention developers or target group). However, there was limited reporting about the barriers or facilitators within specific interventions, partly because many authors did not always share the lessons learned within their interventions. We should note that the barriers and facilitators may not be generalizable across different lifestyle behaviors, and few may apply to all SES groups and not only to eHealth interventions for low SES groups.

The studies examined the effectiveness of eHealth lifestyle interventions and showed promising but inconsistent results. They showed small effects of smoking cessation, nutrition, increased physical activity, and weight loss. These studies provided limited information about which components contributed to the effectiveness of the intervention, making it difficult to conclude why these interventions worked when compared to those that were unsuccessful. This is in line with the results of the systematic review by Kohl et al [90], which found that effect sizes were small, variable, and unsustainable in eHealth lifestyle interventions for different SES populations and concluded that the efficacy of intervention elements were unclear.

### Different Delivery Methods

The results of this review suggest that eHealth lifestyle interventions delivered via different delivery modes (ie, websites, SMS text messages, or apps) or combined with professional personal support seem to be accepted by people with a low SES. However, it is still unclear which delivery method is the most effective for this target group because each delivery mode has its advantages. Danaher et al [91] and Iribarren et al [92] suggested that interventions delivered via text messages may be an attractive option as they are inexpensive, suitable for most mobile phones, and require little user effort. Conversely, interventions delivered via websites or apps provide a visually pleasing option (ie, videos) for communicating the information and make the intervention interactive. However, it is crucial to consider the digital literacy levels of people with a low SES when choosing the delivery method of an intervention. Blended care (combination of face-to-face services with eHealth) offers people with a low SES timely guidance, which can promote engagement and adherence to the intervention. Therefore, we suggest that combinations of varied eHealth delivery modes and face-to-face elements (ie, human coaching) could engage people with a low SES successfully.

### Reaching the Low SES Group

Overall, it was clear from the studies that it was difficult to reach low SES individuals for participation in eHealth interventions, which is typical for this group. Thus, a different approach to reach this group is crucial. For example, studies have been successful in reaching participants with active recruiting strategies, such as face-to-face or personal contact [31]. The personal approach may reduce the distance between intervention staff and potential users, create a sense of security, and increase engagement [93]. Long-term relationships build trust between health professionals and patients, and such an approach is needed to reach people with a low SES [93,94]. Moreover, with this rapport, individuals may perceive health professionals as more credible, especially within ethnic minorities [94]. Another promising strategy is collaboration with the social network of people with a low SES (eg, caregivers, relatives, and experts) [44]. Recent studies identified the importance of using a personal approach and connecting via existing networks (ie, community centers or ambassadors) to successfully recruit low SES populations for lifestyle interventions [20,93]. Furthermore, we found that social media may achieve this goal since it has a broad reach, but the lack of robust evidence makes it difficult to draw firm conclusions. Social media may be particularly effective to reach young people. However, reaching people with a low SES remains challenging as there is no clear reach strategy. A similar pattern of results was obtained in the systematic review by Bonevski et al [95], which found that proven strategies to reach socially disadvantaged groups were rare. This highlights the importance of tailoring reach strategies, both online and offline, to target different types of groups (eg, young populations and ethnic minorities) within the low SES population. Lessons can also be drawn from traditional lifestyle interventions that provide insight into reaching low SES groups [96].

### First Phase of Intervention Design and Co-creation

We noted that few studies based their interventions on behavioral theories. When behavioral theories were reported, authors rarely elaborated on how they applied these theories. These results seem to be consistent with other research that found that behavioral theories were seldom applied in interventions [88,97,98]. One possible explanation for this might be that intervention components are developed with a practical viewpoint in mind or a pre-existing belief in the benefit of these components, since they have been used previously in effective interventions [98] Alternatively, it may be that certain behavioral theories were not found to be useful for the development of the intervention at hand and were therefore not applied [99]. However, using theories in interventions has been indicated to increase their effectiveness.

There are several issues to consider in the co-creation of eHealth interventions, such as how and when stakeholders and users get involved. In recent years, more attention has been paid to the role of stakeholders (including users) in public interventions; however, involving stakeholders (eg, people with a low SES and health professionals) from the beginning is time-consuming and expensive [32]. Follow-up research needs to explore the best way to actively involve low SES individuals in developing and evaluating interventions, as co-creating with end users seems promising.

### Implementation

The results of this review show that the development, evaluation, and implementation of eHealth are difficult to distinguish from each other and that the implementation of the intervention takes place during its development. As advocated by Pieterse et al [100], eHealth development and implementation should be intertwined. Implementation should be accounted for from the start of the development process; this is especially true for people with a low SES, since their characteristics, such as low digital skills, may hinder the interventions’ implementation [61].

A shortage of resources is also known to impact implementation. These findings are directly in line with previous findings. For example, Lau et al [101] and Ross et al [102] found in their reviews that available resources, including time, funding, and staff, can be both barriers and facilitators in the implementation of interventions.

### Recommendation for Design and Research, and Limitations

There are still unanswered questions in the development, reach, use, evaluation, and implementation of eHealth interventions for a low SES population, as the research is in its infancy. Using existing guidelines (eg, the CONSORT checklist) or other frameworks could guide in reporting information comprehensively and clearly [29]. For instance, use of the behavior change technique taxonomy by Michie et al [89] can help researchers to report on the behavioral theories and techniques applied in the intervention. Furthermore, it is vital to report more detailed information on how participants use the components of eHealth interventions, which may help identify elements that contribute to the effectiveness of eHealth interventions. This information could be beneficial for future studies and interventions as it can guide developers in the design and implementation of effective eHealth interventions. Another recommendation is to collaborate with researchers, developers, and stakeholders (including users) in the development, evaluation, and implementation of eHealth lifestyle interventions, to fine-tune these to the target group’s needs and requirements. Involving the social networks (eg, relatives and peers) of low SES participants in eHealth lifestyle interventions also seems promising. Research shows that engaging social networks can support low SES participants who experience problems with their digital skills [60]. It is therefore important to investigate what role social networks should play within eHealth lifestyle interventions. Finally, although many studies advised making the content of eHealth interventions accessible to people with low skills, clear recommendations for developers and researchers on how eHealth interventions for low SES populations can be developed, implemented, and evaluated were lacking. Future research should focus on how we can devise holistic eHealth guidelines that can assist developers and researchers with the creation of eHealth interventions that take the capabilities and requirements of this target group into account.

This review is the first to focus on state-of-the-art available knowledge about developing and evaluating eHealth lifestyle interventions, and reaching people with a low SES to realize behavioral change and improve health in these people. The barriers and facilitators that we found offer promising elements that eHealth developers can use as a toolbox to connect eHealth with low SES target groups. Further research on the method of using these tools is still needed. However, this review has some limitations. First, we only included studies on eHealth interventions that focused on lifestyle behaviors and excluded studies on interventions aimed at other relevant areas for low SES individuals (mental health, and medical, legal, and financial issues). These interventions may provide additional insights. Second, as the primary focus was to gain insight into how eHealth lifestyle interventions are developed and evaluated for low SES individuals, we did not assess the quality of the studies and their results (ie, systematic review). Finally, we focused on the low SES group in general and did not distinguish between subgroups. Although ethnicity is not an indicator of SES, ethnic minorities (eg, non-Western immigrants and African American individuals) were often mentioned as prominent groups in the studies. It is therefore important to consider the differences within the low SES population, with the aim of not further increasing health disparities.

### Conclusions

This scoping review provides an overview of the available scientific knowledge on the behavioral and technological components, barriers, and facilitators in the development, evaluation, and implementation of eHealth lifestyle interventions. Although eHealth intervention development is diverse, contributing to the varying results in this review, certain factors may be beneficial for building and using eHealth interventions and reaching people with a low SES. Iterative design of interventions, use of visual and multimedia elements, and social support seem to be important facilitators for eHealth interventions. Technical challenges using eHealth interventions, lack of time in low SES groups, and limited resources appear to be key barriers for eHealth interventions. Understanding these barriers and facilitators may generate insights into how to optimize eHealth interventions for people with a low SES. Developing eHealth interventions for people with a low SES requires consideration of their specific needs and characteristics, and the involvement of users. This may contribute to the use of interventions and may facilitate their implementation.

Guidelines should be developed to aid stakeholders in developing and evaluating eHealth interventions. Moreover, high-quality studies are needed to investigate how eHealth lifestyle interventions can be customized to meet the needs of participants with a low SES. Future studies could benefit significantly from detailed reporting on eHealth interventions for this target group.

## Appendix

Multimedia Appendix 1 Full search strings per database.

Multimedia Appendix 2 Key constructs and definitions for data extraction.

Multimedia Appendix 3 Overview of included publications (characteristic studies and theories).

## Abbreviations

CeHRes: Center for eHealth Research
RCT: randomized controlled trial
RE-AIM: reach, effectiveness, adoption, implementation, and maintenance
SES: socioeconomic status

## References

[1] (2020). The top 10 causes of death. World Health Organization.

[2] Artinian NT, Fletcher GF, Mozaffarian D, Kris-Etherton P, Van Horn L, Lichtenstein AH, Kumanyika S, Kraus WE, Fleg JL, Redeker NS, Meininger JC, Banks J, Stuart-Shor EM, Fletcher BJ, Miller TD, Hughes S, Braun LT, Kopin LA, Berra K, Hayman LL, Ewing LJ, Ades PA, Durstine JL, Houston-Miller N, Burke LE, American Heart Association Prevention Committee of the Council on Cardiovascular Nursing (2010). Interventions to promote physical activity and dietary lifestyle changes for cardiovascular risk factor reduction in adults: a scientific statement from the American Heart Association. Circulation.

[3] Lawrence M, Kerr S, McVey C, Godwin J (2012). The effectiveness of secondary prevention lifestyle interventions designed to change lifestyle behavior following stroke: summary of a systematic review. Int J Stroke.

[4] Brown T, Avenell A, Edmunds LD, Moore H, Whittaker V, Avery L, Summerbell C (2009). Systematic review of long-term lifestyle interventions to prevent weight gain and morbidity in adults. Obes Rev.

[5] Stringhini S, Carmeli C, Jokela M, Avendaño M, Muennig P, Guida F, Ricceri F, d'Errico A, Barros H, Bochud M, Chadeau-Hyam M, Clavel-Chapelon F, Costa G, Delpierre C, Fraga S, Goldberg M, Giles GG, Krogh V, Kelly-Irving M, Layte R, Lasserre AM, Marmot MG, Preisig M, Shipley MJ, Vollenweider P, Zins M, Kawachi I, Steptoe A, Mackenbach JP, Vineis P, Kivimäki M, LIFEPATH consortium (2017). Socioeconomic status and the 25 × 25 risk factors as determinants of premature mortality: a multicohort study and meta-analysis of 1·7 million men and women. Lancet.

[6] Di Girolamo C, Nusselder WJ, Bopp M, Brønnum-Hansen H, Costa G, Kovács K, Leinsalu M, Martikainen P, Pacelli B, Rubio Valverde J, Mackenbach JP (2020). Progress in reducing inequalities in cardiovascular disease mortality in Europe. Heart.

[7] Michie S, Jochelson K, Markham WA, Bridle C (2009). Low-income groups and behaviour change interventions: a review of intervention content, effectiveness and theoretical frameworks. J Epidemiol Community Health.

[8] Mackenbach JP, Valverde JR, Bopp M, Brønnum-Hansen H, Deboosere P, Kalediene R, Kovács K, Leinsalu M, Martikainen P, Menvielle G, Regidor E, Nusselder WJ (2019). Determinants of inequalities in life expectancy: an international comparative study of eight risk factors. Lancet Public Health.

[9] Macintyre A, Ferris D, Gonçalves B, Quinn N (2018). What has economics got to do with it? The impact of socioeconomic factors on mental health and the case for collective action. Palgrave Commun.

[10] Silva M, Loureiro A, Cardoso G (2016). Social determinants of mental health: A review of the evidence. The European Journal of Psychiatry.

[11] Bull ER, Dombrowski SU, McCleary N, Johnston M (2014). Are interventions for low-income groups effective in changing healthy eating, physical activity and smoking behaviours? A systematic review and meta-analysis. BMJ Open.

[12] Bukman AJ, Teuscher D, Feskens EJM, van Baak MA, Meershoek A, Renes RJ (2014). Perceptions on healthy eating, physical activity and lifestyle advice: opportunities for adapting lifestyle interventions to individuals with low socioeconomic status. BMC Public Health.

[13] Coupe N, Cotterill S, Peters S (2018). Tailoring lifestyle interventions to low socio-economic populations: a qualitative study. BMC Public Health.

[14] Sheehy-Skeffington J (2020). The effects of low socioeconomic status on decision-making processes. Curr Opin Psychol.

[15] Reinwand DA, Schulz DN, Crutzen R, Kremers SP, de Vries H (2015). Who Follows eHealth Interventions as Recommended? A Study of Participants' Personal Characteristics From the Experimental Arm of a Randomized Controlled Trial. J Med Internet Res.

[16] Ossebaard HC, Van Gemert-Pijnen L (2016). eHealth and quality in health care: implementation time. Int J Qual Health Care.

[17] Lustria MLA, Noar SM, Cortese J, Van Stee SK, Glueckauf RL, Lee J (2013). A meta-analysis of web-delivered tailored health behavior change interventions. J Health Commun.

[18] Olson CM (2016). Behavioral Nutrition Interventions Using e- and m-Health Communication Technologies: A Narrative Review. Annu Rev Nutr.

[19] Crutzen R, van der Vaart R, Evers A, Bode C, van Gemert-Pijnen L, Kelders SM, Kip H, Sanderman R (2018). Public health, behavioural medicine and eHealth technology. eHealth Research, Theory and Development: A Multidisciplinary Approach.

[20] Faber JS, Al-Dhahir I, Reijnders T, Chavannes NH, Evers AWM, Kraal JJ, van den Berg-Emons HJG, Visch VT (2021). Attitudes Toward Health, Healthcare, and eHealth of People With a Low Socioeconomic Status: A Community-Based Participatory Approach. Front Digit Health.

[21] Van Velsen L, Wentzel J, Van Gemert-Pijnen JE (2013). Designing eHealth that Matters via a Multidisciplinary Requirements Development Approach. JMIR Res Protoc.

[22] Latulippe K, Hamel C, Giroux D (2017). Social Health Inequalities and eHealth: A Literature Review With Qualitative Synthesis of Theoretical and Empirical Studies. J Med Internet Res.

[23] Brown J, Michie S, Geraghty AWA, Yardley L, Gardner B, Shahab L, Stapleton JA, West R (2014). Internet-based intervention for smoking cessation (StopAdvisor) in people with low and high socioeconomic status: a randomised controlled trial. Lancet Respir Med.

[24] Hayba N, Partridge SR, Nour MM, Grech A, Allman Farinelli M (2018). Effectiveness of lifestyle interventions for preventing harmful weight gain among young adults from lower socioeconomic status and ethnically diverse backgrounds: a systematic review. Obes Rev.

[25] (2019). Recommendations on digital interventions for health system strengthening. World Health Organization.

[26] Arksey H, O'Malley L (2005). Scoping studies: towards a methodological framework. International Journal of Social Research Methodology.

[27] van Gemert-Pijnen JE, Nijland N, van Limburg M, Ossebaard HC, Kelders SM, Eysenbach G, Seydel ER (2011). A holistic framework to improve the uptake and impact of eHealth technologies. J Med Internet Res.

[28] Glasgow RE, Harden SM, Gaglio B, Rabin B, Smith ML, Porter GC, Ory MG, Estabrooks PA (2019). RE-AIM Planning and Evaluation Framework: Adapting to New Science and Practice With a 20-Year Review. Front Public Health.

[29] Eysenbach G, CONSORT-EHEALTH Group (2011). CONSORT-EHEALTH: improving and standardizing evaluation reports of Web-based and mobile health interventions. J Med Internet Res.

[30] Wayne N, Perez DF, Kaplan DM, Ritvo P (2015). Health Coaching Reduces HbA1c in Type 2 Diabetic Patients From a Lower-Socioeconomic Status Community: A Randomized Controlled Trial. J Med Internet Res.

[31] Stanczyk NE, Crutzen R, Bolman C, Muris J, de Vries H (2013). Influence of delivery strategy on message-processing mechanisms and future adherence to a Dutch computer-tailored smoking cessation intervention. J Med Internet Res.

[32] Springvloet L, Lechner L, de Vries H, Candel MJJM, Oenema A (2015). Short- and medium-term efficacy of a Web-based computer-tailored nutrition education intervention for adults including cognitive and environmental feedback: randomized controlled trial. J Med Internet Res.

[33] Schneider F, van Osch L, Schulz DN, Kremers SP, de Vries H (2012). The influence of user characteristics and a periodic email prompt on exposure to an internet-delivered computer-tailored lifestyle program. J Med Internet Res.

[34] Ramirez M, Wu S (2017). Phone Messaging to Prompt Physical Activity and Social Support Among Low-Income Latino Patients With Type 2 Diabetes: A Randomized Pilot Study. JMIR Diabetes.

[35] Herring SJ, Cruice JF, Bennett GG, Davey A, Foster GD (2014). Using technology to promote postpartum weight loss in urban, low-income mothers: a pilot randomized controlled trial. J Nutr Educ Behav.

[36] Golsteijn RHJ, Bolman C, Peels DA, Volders E, de Vries H, Lechner L (2017). A Web-Based and Print-Based Computer-Tailored Physical Activity Intervention for Prostate and Colorectal Cancer Survivors: A Comparison of User Characteristics and Intervention Use. J Med Internet Res.

[37] Foley P, Steinberg D, Levine E, Askew S, Batch BC, Puleo EM, Svetkey LP, Bosworth HB, DeVries A, Miranda H, Bennett GG (2016). Track: A randomized controlled trial of a digital health obesity treatment intervention for medically vulnerable primary care patients. Contemp Clin Trials.

[38] Delrahim-Howlett K, Chambers CD, Clapp JD, Xu R, Duke K, Moyer RJ, Van Sickle D (2011). Web-based assessment and brief intervention for alcohol use in women of childbearing potential: a report of the primary findings. Alcohol Clin Exp Res.

[39] Clarke P, Evans SH, Neffa-Creech D (2019). Mobile app increases vegetable-based preparations by low-income household cooks: a randomized controlled trial. Public Health Nutr.

[40] Simons D, De Bourdeaudhuij I, Clarys P, De Cocker K, Vandelanotte C, Deforche B (2018). Effect and Process Evaluation of a Smartphone App to Promote an Active Lifestyle in Lower Educated Working Young Adults: Cluster Randomized Controlled Trial. JMIR Mhealth Uhealth.

[41] Silk KJ, Sherry J, Winn B, Keesecker N, Horodynski MA, Sayir A (2008). Increasing nutrition literacy: testing the effectiveness of print, web site, and game modalities. J Nutr Educ Behav.

[42] Silfee VJ, Lopez-Cepero A, Lemon SC, Estabrook B, Nguyen O, Rosal MC (2019). Recruiting low-income postpartum women into two weight loss interventions: in-person versus Facebook delivery. Transl Behav Med.

[43] Griffin JB, Struempler B, Funderburk K, Parmer SM, Tran C, Wadsworth DD (2018). My Quest, an Intervention Using Text Messaging to Improve Dietary and Physical Activity Behaviors and Promote Weight Loss in Low-Income Women. J Nutr Educ Behav.

[44] van Dongen JM, van Poppel MNM, Milder IEJ, van Oers HAM, Brug J (2012). Exploring the reach and program use of Hello World, an email-based health promotion program for pregnant women in the Netherlands. BMC Res Notes.

[45] Cavallo DN, Sisneros JA, Ronay AA, Robbins CL, Jilcott Pitts SB, Keyserling TC, Ni A, Morrow J, Vu MB, Johnston LF, Samuel-Hodge CD (2016). Assessing the Feasibility of a Web-Based Weight Loss Intervention for Low-Income Women of Reproductive Age: A Pilot Study. JMIR Res Protoc.

[46] Brown J, Michie S, Geraghty AWA, Miller S, Yardley L, Gardner B, Shahab L, Stapleton JA, West R (2012). A pilot study of StopAdvisor: a theory-based interactive internet-based smoking cessation intervention aimed across the social spectrum. Addict Behav.

[47] Cavallo DN, Martinez R, Webb Hooper M, Flocke S (2021). Feasibility of a social media-based weight loss intervention designed for low-SES adults. Transl Behav Med.

[48] Griffin JB, Struempler B, Funderburk K, Parmer SM, Tran C, Wadsworth DD (2020). My Quest, a Community-Based mHealth Intervention to Increase Physical Activity and Promote Weight Loss in Predominantly Rural-Dwelling, Low-Income, Alabama Women. Fam Community Health.

[49] Lepore SJ, Collins BN, Killam HW, Barry B (2021). Supportive Accountability and Mobile App Use in a Tobacco Control Intervention Targeting Low-Income Minority Mothers Who Smoke: Observational Study. JMIR Mhealth Uhealth.

[50] Tagai EK, Miller SM, Belfiglio A, Xu J, Wen KY, Hernandez E (2020). Persistent Barriers to Smoking Cessation Among Urban, Underserved Women: A Feasibility Study of Tailored Barriers Text Messages. Matern Child Health J.

[51] Kendzor DE, Businelle MS, Waring JJC, Mathews AJ, Geller DW, Barton JM, Alexander AC, Hébert ET, Ra CK, Vidrine DJ (2020). Automated Mobile Delivery of Financial Incentives for Smoking Cessation Among Socioeconomically Disadvantaged Adults: Feasibility Study. JMIR Mhealth Uhealth.

[52] Lohse B (2013). Facebook is an effective strategy to recruit low-income women to online nutrition education. J Nutr Educ Behav.

[53] Radhakrishnan K, Toprac P, O'Hair M, Bias R, Kim MT, Bradley P, Mackert M (2016). Interactive Digital e-Health Game for Heart Failure Self-Management: A Feasibility Study. Games Health J.

[54] Greene EM, O'Brien EC, Kennelly MA, O'Brien OA, Lindsay KL, McAuliffe FM (2021). Acceptability of the Pregnancy, Exercise, and Nutrition Research Study With Smartphone App Support (PEARS) and the Use of Mobile Health in a Mixed Lifestyle Intervention by Pregnant Obese and Overweight Women: Secondary Analysis of a Randomized Controlled Trial. JMIR Mhealth Uhealth.

[55] Michie S, Brown J, Geraghty AWA, Miller S, Yardley L, Gardner B, Shahab L, McEwen A, Stapleton JA, West R (2012). Development of StopAdvisor: A theory-based interactive internet-based smoking cessation intervention. Transl Behav Med.

[56] Silfee VJ, Lopez-Cepero A, Lemon SC, Estabrook B, Nguyen O, Wang ML, Rosal MC (2018). Adapting a Behavioral Weight Loss Intervention for Delivery via Facebook: A Pilot Series Among Low-Income Postpartum Women. JMIR Form Res.

[57] Lohse B, Arnold K, Wamboldt P (2013). Evaluation of About Being Active, an online lesson about physical activity shows that perception of being physically active is higher in eating competent low-income women. BMC Womens Health.

[58] Simons D, De Bourdeaudhuij I, Clarys P, De Cocker K, Vandelanotte C, Deforche B (2018). A Smartphone App to Promote an Active Lifestyle in Lower-Educated Working Young Adults: Development, Usability, Acceptability, and Feasibility Study. JMIR Mhealth Uhealth.

[59] Spears CA, Bell SA, Scarlett CA, Anderson NK, Cottrell-Daniels C, Lotfalian S, Bandlamudi M, Grant A, Sigurdardottir A, Carter BP, Abroms LC, Wetter DW (2019). Text Messaging to Enhance Mindfulness-Based Smoking Cessation Treatment: Program Development Through Qualitative Research. JMIR Mhealth Uhealth.

[60] Régnier F, Dugré M, Darcel N, Adamiec C (2018). Providing a Smart Healthy Diet for the Low-Income Population: Qualitative Study on the Usage and Perception of a Designed Cooking App. JMIR Mhealth Uhealth.

[61] Fontil V, McDermott K, Tieu L, Rios C, Gibson E, Sweet CC, Payne M, Lyles CR (2016). Adaptation and Feasibility Study of a Digital Health Program to Prevent Diabetes among Low-Income Patients: Results from a Partnership between a Digital Health Company and an Academic Research Team. J Diabetes Res.

[62] Evans SH, Clarke P (2019). Resolving design issues in developing a nutrition app: A case study using formative research. Eval Program Plann.

[63] Leak TM, Benavente L, Goodell LS, Lassiter A, Jones L, Bowen S (2014). EFNEP graduates' perspectives on social media to supplement nutrition education: focus group findings from active users. J Nutr Educ Behav.

[64] Carolan-Olah M, Vasilevski V, Nagle C, Stepto N (2021). Overview of a new eHealth intervention to promote healthy eating and exercise in pregnancy: Initial user responses and acceptability. Internet Interv.

[65] Bond MH, Bunge EL, Leykin Y, Barrera AZ, Wickham RE, Barlow MR, Reyes S, Pineda B, Ceja AM, Cano M, Muñoz RF (2021). Development and usability of a Spanish/English smoking cessation website: lessons learned. Mhealth.

[66] Atkinson NL, Saperstein SL, Desmond SM, Gold RS, Billing AS, Tian J (2009). Rural eHealth nutrition education for limited-income families: an iterative and user-centered design approach. J Med Internet Res.

[67] Flaherty SJ, McCarthy MB, Collins AM, McCafferty C, McAuliffe FM (2020). A phenomenological exploration of change towards healthier food purchasing behaviour in women from a lower socioeconomic background using a health app. Appetite.

[68] Kothari A, Godleski S, Abu B (2020). Mobile-based consortium of parenting resources for low-income and underserved mothers and caregivers: app development, testing and lessons learned. Health and Technology.

[69] Yee L, Taylor S, Young M, Williams M, Niznik C, Simon M (2020). Evaluation of a Text Messaging Intervention to Support Self-Management of Diabetes During Pregnancy Among Low-Income, Minority Women: Qualitative Study. JMIR Diabetes.

[70] Whittemore R, Vilar-Compte M, Burrola-Méndez S, Lozano-Marrufo A, Delvy R, Pardo-Carrillo M, De La Cerda S, Pena-Purcell N, Pérez-Escamilla R (2020). Development of a diabetes self-management + mHealth program: tailoring the intervention for a pilot study in a low-income setting in Mexico. Pilot Feasibility Stud.

[71] Aldoory L, Yaros RA, Prado AA, Roberts E, Briones RL (2016). Piloting Health Text Messages for Rural Low-Income Mothers: Effects of Source Similarity and Simple Action Steps. Health Promot Pract.

[72] Athavale P, Thomas M, Delgadillo-Duenas AT, Leong K, Najmabadi A, Harleman E, Rios C, Quan J, Soria C, Handley MA (2016). Linking High Risk Postpartum Women with a Technology Enabled Health Coaching Program to Reduce Diabetes Risk and Improve Wellbeing: Program Description, Case Studies, and Recommendations for Community Health Coaching Programs. J Diabetes Res.

[73] Garvin TM, Chiappone A, Boyd L, Stern K, Panichelli J, Edwards Hall LA, Yaroch AL (2019). Cooking Matters Mobile Application: a meal planning and preparation tool for low-income parents. Public Health Nutr.

[74] Mayberry LS, Berg CA, Harper KJ, Osborn CY (2016). The Design, Usability, and Feasibility of a Family-Focused Diabetes Self-Care Support mHealth Intervention for Diverse, Low-Income Adults with Type 2 Diabetes. J Diabetes Res.

[75] Pathak LE, Aguilera A, Williams JJ, Lyles CR, Hernandez-Ramos R, Miramontes J, Cemballi AG, Figueroa CA (2021). Developing Messaging Content for a Physical Activity Smartphone App Tailored to Low-Income Patients: User-Centered Design and Crowdsourcing Approach. JMIR Mhealth Uhealth.

[76] Stotz S, Lee JS (2018). Development of an Online Smartphone-Based eLearning Nutrition Education Program for Low-Income Individuals. J Nutr Educ Behav.

[77] Tabak RG, Strickland JR, Stein RI, Dart H, Colditz GA, Kirk B, Dale AM, Evanoff BA (2018). Development of a scalable weight loss intervention for low-income workers through adaptation of interactive obesity treatment approach (iOTA). BMC Public Health.

[78] Wayne N, Ritvo P (2014). Smartphone-enabled health coach intervention for people with diabetes from a modest socioeconomic strata community: single-arm longitudinal feasibility study. J Med Internet Res.

[79] Neuenschwander LM, Abbott A, Mobley AR (2013). Comparison of a web-based vs in-person nutrition education program for low-income adults. J Acad Nutr Diet.

[80] Burner E, Zhang M, Terp S, Ford Bench K, Lee J, Lam CN, Torres JR, Menchine M, Arora S (2020). Feasibility and Acceptability of a Text Message-Based Intervention to Reduce Overuse of Alcohol in Emergency Department Patients: Controlled Proof-of-Concept Trial. JMIR Mhealth Uhealth.

[81] Brunette MF, Gunn W, Alvarez H, Finn PC, Geiger P, Ferron JC, McHugo GJ (2015). A pre-post pilot study of a brief, web-based intervention to engage disadvantaged smokers into cessation treatment. Addict Sci Clin Pract.

[82] Stanczyk NE, Bolman C, Smit ES, Candel MJJM, Muris JWM, de Vries H (2014). How to encourage smokers to participate in web-based computer-tailored smoking cessation programs: a comparison of different recruitment strategies. Health Educ Res.

[83] Aguilera A, Figueroa CA, Hernandez-Ramos R, Sarkar U, Cemballi A, Gomez-Pathak L, Miramontes J, Yom-Tov E, Chakraborty B, Yan X, Xu J, Modiri A, Aggarwal J, Jay Williams J, Lyles CR (2020). mHealth app using machine learning to increase physical activity in diabetes and depression: clinical trial protocol for the DIAMANTE Study. BMJ Open.

[84] van Dijk W, Oosterman M, Jansen I, de Vente W, Huizink A (2021). Stress- and smoke free pregnancy study protocol: a randomized controlled trial of a personalized eHealth intervention including heart rate variability-biofeedback to support pregnant women quit smoking via stress reduction. BMC Public Health.

[85] Golsteijn RHJ, Bolman C, Volders E, Peels DA, de Vries H, Lechner L (2017). Development of a computer-tailored physical activity intervention for prostate and colorectal cancer patients and survivors: OncoActive. BMC Cancer.

[86] Kim SE, Castro Sweet CM, Gibson E, Madero EN, Rubino B, Morrison J, Rosen D, Imberg W, Cousineau MR (2018). Evaluation of a digital diabetes prevention program adapted for the Medicaid population: Study design and methods for a non-randomized, controlled trial. Contemp Clin Trials Commun.

[87] Patten CA, Fu S, Vickerman K, Bock MJ, Nelson D, Zhu S, Balls-Berry JE, Torres AJ, Brockman TA, Hughes CA, Klein AE, Valdez-Soto M, Keller PA (2019). Support person interventions to increase use of quitline services among racially diverse low-income smokers: A pilot study. Addict Behav Rep.

[88] Michie S, Yardley L, West R, Patrick K, Greaves F (2017). Developing and Evaluating Digital Interventions to Promote Behavior Change in Health and Health Care: Recommendations Resulting From an International Workshop. J Med Internet Res.

[89] Michie S, Richardson M, Johnston M, Abraham C, Francis J, Hardeman W, Eccles MP, Cane J, Wood CE (2013). The behavior change technique taxonomy (v1) of 93 hierarchically clustered techniques: building an international consensus for the reporting of behavior change interventions. Ann Behav Med.

[90] Kohl LFM, Crutzen R, de Vries NK (2013). Online prevention aimed at lifestyle behaviors: a systematic review of reviews. J Med Internet Res.

[91] Danaher BG, Brendryen H, Seeley JR, Tyler MS, Woolley T (2015). From black box to toolbox: Outlining device functionality, engagement activities, and the pervasive information architecture of mHealth interventions. Internet Interv.

[92] Iribarren SJ, Cato K, Falzon L, Stone PW (2017). What is the economic evidence for mHealth? A systematic review of economic evaluations of mHealth solutions. PLoS One.

[93] Stuber JM, Middel CNH, Mackenbach JD, Beulens JWJ, Lakerveld J (2020). Successfully Recruiting Adults with a Low Socioeconomic Position into Community-Based Lifestyle Programs: A Qualitative Study on Expert Opinions. Int J Environ Res Public Health.

[94] Bukman AJ, Teuscher D, Ben Meftah J, Groenenberg I, Crone MR, van Dijk S, Bos MB, Feskens EJM (2016). Exploring strategies to reach individuals of Turkish and Moroccan origin for health checks and lifestyle advice: a mixed-methods study. BMC Fam Pract.

[95] Bonevski B, Randell M, Paul C, Chapman K, Twyman L, Bryant J, Brozek I, Hughes C (2014). Reaching the hard-to-reach: a systematic review of strategies for improving health and medical research with socially disadvantaged groups. BMC Med Res Methodol.

[96] van den Brand FA, Magnée T, de Haan-Bouma L, Barendregt C, Chavannes NH, van Schayck OCP, Nagelhout GE (2019). Implementation of Financial Incentives for Successful Smoking Cessation in Real-Life Company Settings: A Qualitative Needs Assessment among Employers. Int J Environ Res Public Health.

[97] Webb T, Joseph J, Yardley L, Michie S (2010). Using the internet to promote health behavior change: a systematic review and meta-analysis of the impact of theoretical basis, use of behavior change techniques, and mode of delivery on efficacy. J Med Internet Res.

[98] Parker S, Prince A, Thomas L, Song H, Milosevic D, Harris MF, IMPACT Study Group (2018). Electronic, mobile and telehealth tools for vulnerable patients with chronic disease: a systematic review and realist synthesis. BMJ Open.

[99] Riley W, Rivera D (2014). Methodologies for optimizing behavioral interventions: introduction to special section. Transl Behav Med.

[100] Pieterse M, Kip H, Cruz-Martínez R, van Gemert-Pijnen L, Kelders SM, Kip H, Sanderman R (2018). The complexity of eHealth implementation: A theoretical and practical perspective. eHealth Research, Theory and Development: A Multidisciplinary Approach.

[101] Lau R, Stevenson F, Ong BN, Dziedzic K, Treweek S, Eldridge S, Everitt H, Kennedy A, Qureshi N, Rogers A, Peacock R, Murray E (2016). Achieving change in primary care--causes of the evidence to practice gap: systematic reviews of reviews. Implement Sci.

[102] Ross J, Stevenson F, Lau R, Murray E (2016). Factors that influence the implementation of e-health: a systematic review of systematic reviews (an update). Implement Sci.

