# Supplementary material for: The Barriers and Facilitators of eHealth-Based Lifestyle Intervention Programs for People With a Low Socioeconomic Status: Scoping Review
Source: J Med Internet Res. 2022 Aug 24;24(8):e34229. doi: 10.2196/34229 (PMC9453585; doi:10.2196/34229)
Supplement: Multimedia Appendix 1 [file jmir_v24i8e34229_app1.doc]

Multimedia Appendix 1 - Full search strings per database

Databases:

**PubMed**

**(**(("Minority Groups"[majr] OR "Minority Health"[majr] OR "Poverty"[majr] **OR** "deprived communities"[ti] OR "deprived community"[ti] OR "disadvantaged communities"[ti] OR "disadvantaged community"[ti] OR "disadvantaged families"[ti] OR "disadvantaged family"[ti] OR "Economic Status"[ti] OR "high-risk families"[ti] OR "high-risk family"[ti] OR "low educated"[ti] OR "low education"[ti] OR "low educational"[ti] OR "low income"[ti] OR "low incomes"[ti] OR "low social"[ti] OR "low socio"[ti] OR "low socio economic"[ti] OR "lower educated"[ti] OR "lower education"[ti] OR "lower educational"[ti] OR "lower income"[ti] OR "lower incomes"[ti] OR "lower social"[ti] OR "lower socio economic"[ti] OR "lowest educated"[ti] OR "lowest education"[ti] OR "lowest educational"[ti] OR "lowest income"[ti] OR "lowest incomes"[ti] OR "lowest social"[ti] OR "minority communities"[ti] OR "minority community"[ti] OR "minority group"[ti] OR "minority groups"[ti] OR "minority health"[ti] OR "Poverty"[ti] OR "SES"[ti] OR "Social Class"[ti] OR "Social Classes"[ti] OR "socioeconomic disparities"[ti] OR "socio-economic disparities"[ti] OR "socioeconomic disparity"[ti] OR "socio-economic disparity"[ti] OR "vulnerable"[ti] OR "vulnerables"[ti] OR "Social Health Inequalities"[ti] OR "Social Health Inequality"[ti] OR "Social Inequalities"[ti] OR "Social Inequality"[ti] OR "Healthcare Disparities"[majr]) AND ("Therapy, computer-assisted"[mesh:noexp] OR "computer-assisted therapy"[tiab] OR "computer assisted therapy"[tiab] OR "computer-assisted"[tiab] OR "computer assisted"[tiab] OR "online therapy"[tiab] OR computer application*[tiab] OR "Electronic Learning"[tiab] OR "Computer Mediated Communication"[tiab] OR "Computer Mediated Communications"[tiab] OR "Internet"[mesh] OR "internet"[tiab] OR "Electronic mail"[mesh] OR "electronic mail"[tiab] OR "e mail"[tiab] OR "e-mail"[tiab] OR "email"[tiab] OR "Telemedicine"[mesh] OR "telemedicine"[tiab] OR telemed*[tiab] OR "remote"[tiab] OR "web-based"[tiab] OR "webbased"[tiab] OR "web based"[tiab] OR "e-health"[tiab] OR "e health"[tiab] OR "ehealth"[tiab] OR "m-health"[tiab] OR "m health"[tiab] OR "mhealth"[tiab] OR "mobile health"[tiab] OR "mobile"[tiab] OR "online"[tiab] OR "on-line"[tiab] OR "on line"[tiab] OR "digital"[tiab] OR "telehealth"[tiab] OR "tele-health"[tiab] OR "tele health"[tiab] OR "iCBT"[tiab] OR "mobile application"[tiab] OR "Mobile applications"[mesh] OR "mobile applications"[tiab] OR "mobile application"[tiab] OR "mobile apps"[tiab] OR "mobile app"[tiab] OR e-consult*[tiab] OR econsult*[tiab] OR "remote communication"[tiab] OR "remote computer"[tiab] OR "remote computers"[tiab] OR "remote consultation"[tiab] OR "remote health care"[tiab] OR "remote healthcare"[tiab] OR "remote monitoring"[tiab] OR "remote system"[tiab] OR "remote systems"[tiab] OR "remote technologies"[tiab] OR "remote technology"[tiab] OR "teleconsultation"[tiab] OR teleconsult*[tiab] OR "smart technology"[tiab] OR smart technol*[tiab] OR "wearable technology"[tiab] OR "wearable technologies"[tiab] OR "Telephone"[mesh] OR telephon*[tiab] OR "phone"[tiab] OR "phones"[tiab] OR "Cell Phones"[tiab] OR "Smartphone"[tiab] OR "Cell Phone"[tiab] OR "cellular phone"[tiab] OR "cellular phones"[tiab] OR "Smartphones"[tiab] OR iphon*[tiab] OR "Text Messaging"[tiab] OR text messag*[tiab] OR "texting"[tiab] OR "short message service"[tiab] OR "SMS"[tiab] OR "app"[tiab] OR "apps"[tiab] OR webapp*[tiab] OR "mass communication"[tiab] OR "blogging"[tiab] OR "blog"[tiab] OR "weblog"[tiab] OR "social media"[tiab] OR twitter*[tiab] OR facebook*[tiab] OR webcast*[tiab] OR "Webcasts as Topic"[mesh] OR "Telecommunications"[mesh] OR web portal*[tiab] OR electronic communication*[tiab] OR "tele-care"[tiab] OR "telecare"[tiab] OR "tele-monitoring"[tiab] OR "telemonitoring"[tiab] OR "website"[tiab] OR "websites"[tiab] OR "wireless"[tiab] OR "personal digital assistant"[tiab] OR "computer-assisted instruction"[tiab] OR "social network"[tiab] OR social network*[tiab] OR "ipad"[tiab] OR ipad*[tiab] OR "telenursing"[tiab] OR telenurs*[tiab] OR "virtual community"[tiab] OR "webpage"[tiab] OR "webpages"[tiab] OR "web application"[tiab] OR "web applications"[tiab] OR "web access"[tiab] OR "web"[tiab] OR "telerehabilitation"[tiab])) **OR** (("Poverty"[mesh] OR "deprived communities"[tiab] OR "deprived community"[tiab] OR "disadvantaged communities"[tiab] OR "disadvantaged community"[tiab] OR "disadvantaged families"[tiab] OR "disadvantaged family"[tiab] OR "Economic Status"[tiab] OR "high-risk families"[tiab] OR "high-risk family"[tiab] OR "low educated"[tiab] OR "low education"[tiab] OR "low educational"[tiab] OR "low income"[tiab] OR "low incomes"[tiab] OR "low social"[tiab] OR "low socio"[tiab] OR "low socio economic"[tiab] OR "lower educated"[tiab] OR "lower education"[tiab] OR "lower educational"[tiab] OR "lower income"[tiab] OR "lower incomes"[tiab] OR "lower social"[tiab] OR "lower socio economic"[tiab] OR "lowest educated"[tiab] OR "lowest education"[tiab] OR "lowest educational"[tiab] OR "lowest income"[tiab] OR "lowest incomes"[tiab] OR "lowest social"[tiab] OR "minority communities"[tiab] OR "minority community"[tiab] OR "minority group"[tiab] OR "minority groups"[tiab] OR "minority health"[tiab] OR "Poverty"[tiab] OR "SES"[tiab] OR "Social Class"[tiab] OR "Social Classes"[tiab] OR "socioeconomic disparities"[tiab] OR "socio-economic disparities"[tiab] OR "socioeconomic disparity"[tiab] OR "socio-economic disparity"[tiab] OR "vulnerable"[tiab] OR "vulnerables"[tiab] OR "Social Health Inequalities"[tiab] OR "Social Health Inequality"[tiab] OR "Social Inequalities"[tiab] OR "Social Inequality"[tiab] OR "Healthcare Disparities"[mesh]) AND ("Therapy, computer-assisted"[majr:noexp] OR "computer-assisted therapy"[ti] OR "computer assisted therapy"[ti] OR "computer-assisted"[ti] OR "computer assisted"[ti] OR "online therapy"[ti] OR computer application*[ti] OR "Electronic Learning"[ti] OR "Computer Mediated Communication"[ti] OR "Computer Mediated Communications"[ti] OR "Internet"[majr] OR "internet"[ti] OR "Electronic mail"[majr] OR "electronic mail"[ti] OR "e mail"[ti] OR "e-mail"[ti] OR "email"[ti] OR "Telemedicine"[majr] OR "telemedicine"[ti] OR telemed*[ti] OR "remote"[ti] OR "web-based"[ti] OR "webbased"[ti] OR "web based"[ti] OR "e-health"[ti] OR "e health"[ti] OR "ehealth"[ti] OR "m-health"[ti] OR "m health"[ti] OR "mhealth"[ti] OR "mobile health"[ti] OR "mobile"[ti] OR "online"[ti] OR "on-line"[ti] OR "on line"[ti] OR "digital"[ti] OR "telehealth"[ti] OR "tele-health"[ti] OR "tele health"[ti] OR "iCBT"[ti] OR "mobile application"[ti] OR "Mobile applications"[majr] OR "mobile applications"[ti] OR "mobile application"[ti] OR "mobile apps"[ti] OR "mobile app"[ti] OR e-consult*[ti] OR econsult*[ti] OR "remote communication"[ti] OR "remote computer"[ti] OR "remote computers"[ti] OR "remote consultation"[ti] OR "remote health care"[ti] OR "remote healthcare"[ti] OR "remote monitoring"[ti] OR "remote system"[ti] OR "remote systems"[ti] OR "remote technologies"[ti] OR "remote technology"[ti] OR "teleconsultation"[ti] OR teleconsult*[ti] OR "smart technology"[ti] OR smart technol*[ti] OR "wearable technology"[ti] OR "wearable technologies"[ti] OR "Telephone"[majr] OR telephon*[ti] OR "phone"[ti] OR "phones"[ti] OR "Cell Phones"[ti] OR "Smartphone"[ti] OR "Cell Phone"[ti] OR "cellular phone"[ti] OR "cellular phones"[ti] OR "Smartphones"[ti] OR iphon*[ti] OR "Text Messaging"[ti] OR text messag*[ti] OR "texting"[ti] OR "short message service"[ti] OR "SMS"[ti] OR "app"[ti] OR "apps"[ti] OR webapp*[ti] OR "mass communication"[ti] OR "blogging"[ti] OR "blog"[ti] OR "weblog"[ti] OR "social media"[ti] OR twitter*[ti] OR facebook*[ti] OR webcast*[ti] OR "Webcasts as Topic"[majr] OR "Telecommunications"[majr] OR web portal*[ti] OR electronic communication*[ti] OR "tele-care"[ti] OR "telecare"[ti] OR "tele-monitoring"[ti] OR "telemonitoring"[ti] OR "website"[ti] OR "websites"[ti] OR "wireless"[ti] OR "personal digital assistant"[ti] OR "computer-assisted instruction"[ti] OR "social network"[ti] OR social network*[ti] OR "ipad"[ti] OR ipad*[ti] OR "telenursing"[ti] OR telenurs*[ti] OR "virtual community"[ti] OR "webpage"[ti] OR "webpages"[ti] OR "web application"[ti] OR "web applications"[ti] OR "web access"[ti] OR "web"[ti] OR "telerehabilitation"[ti]))**)** AND ("Life style"[mesh] OR "life style"[tiab] OR "lifestyle"[tiab] OR "life-style"[tiab] OR "life styles"[tiab] OR "lifestyles"[tiab] OR "life-styles"[tiab] OR "Health behavior"[mesh] OR "health behaviour"[tiab] OR "health behavior"[tiab] OR "Health promotion"[mesh] OR "health promotion"[tiab] OR "self-management"[tiab] OR "self management"[tiab] OR "Exercise"[mesh] OR "exercise"[tiab] OR "physical activity"[tiab] OR "Diet"[mesh] OR "Diet Therapy"[mesh] OR "diet"[tiab] OR "diets"[tiab] OR "dietary"[tiab] OR "fat"[tiab] OR "salt"[tiab] OR "natrium"[tiab] OR "sodium"[tiab] OR "Dietary Carbohydrates"[Mesh] OR carbohydrate*[tiab] OR "calories"[tiab] OR "Dietary Proteins"[Mesh] OR "proteins"[tiab] OR "fat intake"[tiab] OR "salt intake"[tiab] OR "Eating"[mesh] OR "eating"[tiab] OR "Nutrition Therapy"[Mesh] OR "nutrition"[tiab] OR "Smoking"[mesh] OR "smoking"[tiab] OR "Tobacco use"[mesh] OR "tobacco"[tiab] OR "nicotine"[tiab] OR "cigarettes"[tiab] OR "cigarette"[tiab] OR "Drinking Behavior"[mesh] OR "alcohol drinking"[mesh] OR "Alcohol Abstinence"[tiab] OR "Alcohol Drinking"[tiab] OR "alcohol"[tiab] OR "drinking"[mesh] OR "drinking"[tiab] OR "Sleep"[mesh] OR "sleep"[tiab] OR "sedentary"[tiab] OR "Medication Adherence"[Mesh] OR "Medication Adherence"[tiab] OR "medication"[tiab] OR "Body Weight"[mesh] OR "weight"[tiab] OR "body weight"[tiab] OR "Blood glucose"[mesh] OR "blood glucose"[tiab] OR "BMI"[tiab] OR "Body mass index"[mesh] OR "body mass index"[tiab] OR "overweight"[mesh] OR "overweight"[tiab] OR "obesity"[mesh] OR "obesity"[tiab] OR "obese"[tiab] OR "Blood pressure"[mesh] OR "Hypertension"[Mesh] OR "blood pressure"[tiab] OR "blood pressure determination"[mesh] OR "Cholesterol"[mesh] OR "cholesterol"[tiab] OR "triglycerides"[mesh] OR "triglycerides"[tiab] OR "triglyceride"[tiab] OR "glycated hemoglobin A"[tiab] OR "hemoglobin A1c"[tiab] OR "HbA1c"[tiab] OR "glomerular filtration rate"[tiab] OR "GFR"[tiab] OR "glucoregulation"[tiab] OR "cardiac rehabilitation"[tiab]) NOT (("Infant"[mesh] OR "Child"[mesh] OR "Adolescent"[mesh] OR "Infant"[ti] OR "infants"[ti] OR "Child"[ti] OR "children"[ti] OR pediatr*[ti] OR paediatr*[ti] OR Adolescen*[ti] OR "kids"[ti] OR "Childhood"[ti] OR "youth"[ti] OR "youths"[ti]) NOT "Adult"[Mesh]) AND ("reach"[tiab] OR reach*[tiab] OR "outreach"[tiab] OR outreach*[tiab] OR "Community-Institutional Relations"[Mesh] OR "use"[tiab] OR "uses"[tiab] OR "used"[tiab] OR "utilise"[tiab] OR "utilize"[tiab] OR utilis*[tiab] OR utiliz*[tiab] OR "usage"[tiab] OR usage*[tiab] OR "Patient Participation"[mesh] OR "implementation"[tiab] OR implement*[tiab] OR "Health Plan Implementation"[Mesh] OR "Implementation Science"[Mesh] OR "Marketing"[mesh] OR "Public Relations"[mesh] OR "Program Evaluation"[mesh] OR "development"[tiab] OR "developing"[tiab] OR "develop"[tiab])

**MEDLINE**

**(**((*"Minority Groups"/ OR *"Minority Health"/ OR exp *"Poverty"/ **OR** "deprived communities".ti OR "deprived community".ti OR "disadvantaged communities".ti OR "disadvantaged community".ti OR "disadvantaged families".ti OR "disadvantaged family".ti OR "Economic Status".ti OR "high-risk families".ti OR "high-risk family".ti OR "low educated".ti OR "low education".ti OR "low educational".ti OR "low income".ti OR "low incomes".ti OR "low social".ti OR "low socio".ti OR "low socio economic".ti OR "lower educated".ti OR "lower education".ti OR "lower educational".ti OR "lower income".ti OR "lower incomes".ti OR "lower social".ti OR "lower socio economic".ti OR "lowest educated".ti OR "lowest education".ti OR "lowest educational".ti OR "lowest income".ti OR "lowest incomes".ti OR "lowest social".ti OR "minority communities".ti OR "minority community".ti OR "minority group".ti OR "minority groups".ti OR "minority health".ti OR "Poverty".ti OR "SES".ti OR "Social Class".ti OR "Social Classes".ti OR "socioeconomic disparities".ti OR "socio-economic disparities".ti OR "socioeconomic disparity".ti OR "socio-economic disparity".ti OR "vulnerable".ti OR "vulnerables".ti OR "Social Health Inequalities".ti OR "Social Health Inequality".ti OR "Social Inequalities".ti OR "Social Inequality".ti OR exp *"Healthcare Disparities"/) AND ("Therapy, computer-assisted"/ OR "computer-assisted therapy".ti,ab OR "computer assisted therapy".ti,ab OR "computer-assisted".ti,ab OR "computer assisted".ti,ab OR "online therapy".ti,ab OR computer application*.ti,ab OR "Electronic Learning".ti,ab OR "Computer Mediated Communication".ti,ab OR "Computer Mediated Communications".ti,ab OR exp "Internet"/ OR "internet".ti,ab OR exp "Electronic mail"/ OR "electronic mail".ti,ab OR "e mail".ti,ab OR "e-mail".ti,ab OR "email".ti,ab OR exp "Telemedicine"/ OR "telemedicine".ti,ab OR telemed*.ti,ab OR "remote".ti,ab OR "web-based".ti,ab OR "webbased".ti,ab OR "web based".ti,ab OR "e-health".ti,ab OR "e health".ti,ab OR "ehealth".ti,ab OR "m-health".ti,ab OR "m health".ti,ab OR "mhealth".ti,ab OR "mobile health".ti,ab OR "mobile".ti,ab OR "online".ti,ab OR "on-line".ti,ab OR "on line".ti,ab OR "digital".ti,ab OR "telehealth".ti,ab OR "tele-health".ti,ab OR "tele health".ti,ab OR "iCBT".ti,ab OR "mobile application".ti,ab OR exp "Mobile applications"/ OR "mobile applications".ti,ab OR "mobile application".ti,ab OR "mobile apps".ti,ab OR "mobile app".ti,ab OR e-consult*.ti,ab OR econsult*.ti,ab OR "remote communication".ti,ab OR "remote computer".ti,ab OR "remote computers".ti,ab OR "remote consultation".ti,ab OR "remote health care".ti,ab OR "remote healthcare".ti,ab OR "remote monitoring".ti,ab OR "remote system".ti,ab OR "remote systems".ti,ab OR "remote technologies".ti,ab OR "remote technology".ti,ab OR "teleconsultation".ti,ab OR teleconsult*.ti,ab OR "smart technology".ti,ab OR smart technol*.ti,ab OR "wearable technology".ti,ab OR "wearable technologies".ti,ab OR exp "Telephone"/ OR telephon*.ti,ab OR "phone".ti,ab OR "phones".ti,ab OR "Cell Phones".ti,ab OR "Smartphone".ti,ab OR "Cell Phone".ti,ab OR "cellular phone".ti,ab OR "cellular phones".ti,ab OR "Smartphones".ti,ab OR iphon*.ti,ab OR "Text Messaging".ti,ab OR text messag*.ti,ab OR "texting".ti,ab OR "short message service".ti,ab OR "SMS".ti,ab OR "app".ti,ab OR "apps".ti,ab OR webapp*.ti,ab OR "mass communication".ti,ab OR "blogging".ti,ab OR "blog".ti,ab OR "weblog".ti,ab OR "social media".ti,ab OR twitter*.ti,ab OR facebook*.ti,ab OR webcast*.ti,ab OR exp "Webcasts as Topic"/ OR exp "Telecommunications"/ OR web portal*.ti,ab OR electronic communication*.ti,ab OR "tele-care".ti,ab OR "telecare".ti,ab OR "tele-monitoring".ti,ab OR "telemonitoring".ti,ab OR "website".ti,ab OR "websites".ti,ab OR "wireless".ti,ab OR "personal digital assistant".ti,ab OR "computer-assisted instruction".ti,ab OR "social network".ti,ab OR social network*.ti,ab OR "ipad".ti,ab OR ipad*.ti,ab OR "telenursing".ti,ab OR telenurs*.ti,ab OR "virtual community".ti,ab OR "webpage".ti,ab OR "webpages".ti,ab OR "web application".ti,ab OR "web applications".ti,ab OR "web access".ti,ab OR "web".ti,ab OR "telerehabilitation".ti,ab)) **OR** (("Minority Groups"/ OR "Minority Health"/ OR exp "Poverty"/ OR "deprived communities".ti,ab OR "deprived community".ti,ab OR "disadvantaged communities".ti,ab OR "disadvantaged community".ti,ab OR "disadvantaged families".ti,ab OR "disadvantaged family".ti,ab OR "Economic Status".ti,ab OR "high-risk families".ti,ab OR "high-risk family".ti,ab OR "low educated".ti,ab OR "low education".ti,ab OR "low educational".ti,ab OR "low income".ti,ab OR "low incomes".ti,ab OR "low social".ti,ab OR "low socio".ti,ab OR "low socio economic".ti,ab OR "lower educated".ti,ab OR "lower education".ti,ab OR "lower educational".ti,ab OR "lower income".ti,ab OR "lower incomes".ti,ab OR "lower social".ti,ab OR "lower socio economic".ti,ab OR "lowest educated".ti,ab OR "lowest education".ti,ab OR "lowest educational".ti,ab OR "lowest income".ti,ab OR "lowest incomes".ti,ab OR "lowest social".ti,ab OR "minority communities".ti,ab OR "minority community".ti,ab OR "minority group".ti,ab OR "minority groups".ti,ab OR "minority health".ti,ab OR "Poverty".ti,ab OR "SES".ti,ab OR "Social Class".ti,ab OR "Social Classes".ti,ab OR "socioeconomic disparities".ti,ab OR "socio-economic disparities".ti,ab OR "socioeconomic disparity".ti,ab OR "socio-economic disparity".ti,ab OR "vulnerable".ti,ab OR "vulnerables".ti,ab OR "Social Health Inequalities".ti,ab OR "Social Health Inequality".ti,ab OR "Social Inequalities".ti,ab OR "Social Inequality".ti,ab OR exp "Healthcare Disparities"/) AND (*"Therapy, computer-assisted"/ OR "computer-assisted therapy".ti OR "computer assisted therapy".ti OR "computer-assisted".ti OR "computer assisted".ti OR "online therapy".ti OR computer application*.ti OR "Electronic Learning".ti OR "Computer Mediated Communication".ti OR "Computer Mediated Communications".ti OR exp *"Internet"/ OR "internet".ti OR exp *"Electronic mail"/ OR "electronic mail".ti OR "e mail".ti OR "e-mail".ti OR "email".ti OR exp *"Telemedicine"/ OR "telemedicine".ti OR telemed*.ti OR "remote".ti OR "web-based".ti OR "webbased".ti OR "web based".ti OR "e-health".ti OR "e health".ti OR "ehealth".ti OR "m-health".ti OR "m health".ti OR "mhealth".ti OR "mobile health".ti OR "mobile".ti OR "online".ti OR "on-line".ti OR "on line".ti OR "digital".ti OR "telehealth".ti OR "tele-health".ti OR "tele health".ti OR "iCBT".ti OR "mobile application".ti OR exp *"Mobile applications"/ OR "mobile applications".ti OR "mobile application".ti OR "mobile apps".ti OR "mobile app".ti OR e-consult*.ti OR econsult*.ti OR "remote communication".ti OR "remote computer".ti OR "remote computers".ti OR "remote consultation".ti OR "remote health care".ti OR "remote healthcare".ti OR "remote monitoring".ti OR "remote system".ti OR "remote systems".ti OR "remote technologies".ti OR "remote technology".ti OR "teleconsultation".ti OR teleconsult*.ti OR "smart technology".ti OR smart technol*.ti OR "wearable technology".ti OR "wearable technologies".ti OR exp *"Telephone"/ OR telephon*.ti OR "phone".ti OR "phones".ti OR "Cell Phones".ti OR "Smartphone".ti OR "Cell Phone".ti OR "cellular phone".ti OR "cellular phones".ti OR "Smartphones".ti OR iphon*.ti OR "Text Messaging".ti OR text messag*.ti OR "texting".ti OR "short message service".ti OR "SMS".ti OR "app".ti OR "apps".ti OR webapp*.ti OR "mass communication".ti OR "blogging".ti OR "blog".ti OR "weblog".ti OR "social media".ti OR twitter*.ti OR facebook*.ti OR webcast*.ti OR exp *"Webcasts as Topic"/ OR exp *"Telecommunications"/ OR web portal*.ti OR electronic communication*.ti OR "tele-care".ti OR "telecare".ti OR "tele-monitoring".ti OR "telemonitoring".ti OR "website".ti OR "websites".ti OR "wireless".ti OR "personal digital assistant".ti OR "computer-assisted instruction".ti OR "social network".ti OR social network*.ti OR "ipad".ti OR ipad*.ti OR "telenursing".ti OR telenurs*.ti OR "virtual community".ti OR "webpage".ti OR "webpages".ti OR "web application".ti OR "web applications".ti OR "web access".ti OR "web".ti OR "telerehabilitation".ti))**)** AND ("Life style"/ OR "life style".ti,ab OR "lifestyle".ti,ab OR "life-style".ti,ab OR "life styles".ti,ab OR "lifestyles".ti,ab OR "life-styles".ti,ab OR "Health behavior"/ OR "health behaviour".ti,ab OR "health behavior".ti,ab OR "Health promotion"/ OR "health promotion".ti,ab OR "self-management".ti,ab OR "self management".ti,ab OR "Exercise"/ OR "exercise".ti,ab OR "physical activity".ti,ab OR "Diet"/ OR "Diet Therapy"/ OR "diet".ti,ab OR "diets".ti,ab OR "dietary".ti,ab OR "fat".ti,ab OR "salt".ti,ab OR "natrium".ti,ab OR "sodium".ti,ab OR "Dietary Carbohydrates"/ OR carbohydrate*.ti,ab OR "calories".ti,ab OR "Dietary Proteins"/ OR "proteins".ti,ab OR "fat intake".ti,ab OR "salt intake".ti,ab OR "Eating"/ OR "eating".ti,ab OR "Nutrition Therapy"/ OR "nutrition".ti,ab OR "Smoking"/ OR "smoking".ti,ab OR "Tobacco use"/ OR "tobacco".ti,ab OR "nicotine".ti,ab OR "cigarettes".ti,ab OR "cigarette".ti,ab OR "Drinking Behavior"/ OR "alcohol drinking"/ OR "Alcohol Abstinence".ti,ab OR "Alcohol Drinking".ti,ab OR "alcohol".ti,ab OR "drinking"/ OR "drinking".ti,ab OR "Sleep"/ OR "sleep".ti,ab OR "sedentary".ti,ab OR "Medication Adherence"/ OR "Medication Adherence".ti,ab OR "medication".ti,ab OR "Body Weight"/ OR "weight".ti,ab OR "body weight".ti,ab OR "Blood glucose"/ OR "blood glucose".ti,ab OR "BMI".ti,ab OR "Body mass index"/ OR "body mass index".ti,ab OR "overweight"/ OR "overweight".ti,ab OR "obesity"/ OR "obesity".ti,ab OR "obese".ti,ab OR "Blood pressure"/ OR "Hypertension"/ OR "blood pressure".ti,ab OR "blood pressure determination"/ OR "Cholesterol"/ OR "cholesterol".ti,ab OR "triglycerides"/ OR "triglycerides".ti,ab OR "triglyceride".ti,ab OR "glycated hemoglobin A".ti,ab OR "hemoglobin A1c".ti,ab OR "HbA1c".ti,ab OR "glomerular filtration rate".ti,ab OR "GFR".ti,ab OR "glucoregulation".ti,ab OR "cardiac rehabilitation".ti,ab) NOT (("Infant"/ OR "Child"/ OR "Adolescent"/ OR "Infant".ti OR "infants".ti OR "Child".ti OR "children".ti OR pediatr*.ti OR paediatr*.ti OR Adolescen*.ti OR "kids".ti OR "Childhood".ti OR "youth".ti OR "youths".ti) NOT "Adult"/) AND "use".ti,ab NOT ("reach".ti,ab OR reach*.ti,ab OR "outreach".ti,ab OR outreach*.ti,ab OR exp "Community-Institutional Relations"/ OR "uses".ti,ab OR "used".ti,ab OR "utilise".ti,ab OR "utilize".ti,ab OR utilis*.ti,ab OR utiliz*.ti,ab OR "usage".ti,ab OR usage*.ti,ab OR exp "Patient Participation"/ OR "implementation".ti,ab OR implement*.ti,ab OR exp "Health Plan Implementation"/ OR exp "Implementation Science"/ OR exp "Marketing"/ OR exp "Public Relations"/ OR exp "Program Evaluation"/ OR "development".ti,ab OR "developing".ti,ab OR "develop".ti,ab)

**Embase**

(("Minority Groups".ti,ab OR "Minority Health".ti,ab OR "Poverty".ti,ab **OR** "deprived communities".ti,ab OR "deprived community".ti,ab OR "disadvantaged communities".ti,ab OR "disadvantaged community".ti,ab OR "disadvantaged families".ti,ab OR "disadvantaged family".ti,ab OR "Economic Status".ti,ab OR "high-risk families".ti,ab OR "high-risk family".ti,ab OR "low educated".ti,ab OR "low education".ti,ab OR "low educational".ti,ab OR "low income".ti,ab OR "low incomes".ti,ab OR "low social".ti,ab OR "low socio".ti,ab OR "low socio economic".ti,ab OR "lower educated".ti,ab OR "lower education".ti,ab OR "lower educational".ti,ab OR "lower income".ti,ab OR "lower incomes".ti,ab OR "lower social".ti,ab OR "lower socio economic".ti,ab OR "lowest educated".ti,ab OR "lowest education".ti,ab OR "lowest educational".ti,ab OR "lowest income".ti,ab OR "lowest incomes".ti,ab OR "lowest social".ti,ab OR "minority communities".ti,ab OR "minority community".ti,ab OR "minority group".ti,ab OR "minority groups".ti,ab OR "minority health".ti,ab OR "Poverty".ti,ab OR "SES".ti,ab OR "Social Class".ti,ab OR "Social Classes".ti,ab OR "socioeconomic disparities".ti,ab OR "socio-economic disparities".ti,ab OR "socioeconomic disparity".ti,ab OR "socio-economic disparity".ti,ab OR "vulnerable".ti,ab OR "vulnerables".ti,ab OR "Social Health Inequalities".ti,ab OR "Social Health Inequality".ti,ab OR "Social Inequalities".ti,ab OR "Social Inequality".ti,ab OR "Healthcare Disparities".ti,ab OR "Poverty".ti,ab OR "deprived communities".ti,ab OR "deprived community".ti,ab OR "disadvantaged communities".ti,ab OR "disadvantaged community".ti,ab OR "disadvantaged families".ti,ab OR "disadvantaged family".ti,ab OR "Economic Status".ti,ab OR "high-risk families".ti,ab OR "high-risk family".ti,ab OR "low educated".ti,ab OR "low education".ti,ab OR "low educational".ti,ab OR "low income".ti,ab OR "low incomes".ti,ab OR "low social".ti,ab OR "low socio".ti,ab OR "low socio economic".ti,ab OR "lower educated".ti,ab OR "lower education".ti,ab OR "lower educational".ti,ab OR "lower income".ti,ab OR "lower incomes".ti,ab OR "lower social".ti,ab OR "lower socio economic".ti,ab OR "lowest educated".ti,ab OR "lowest education".ti,ab OR "lowest educational".ti,ab OR "lowest income".ti,ab OR "lowest incomes".ti,ab OR "lowest social".ti,ab OR "minority communities".ti,ab OR "minority community".ti,ab OR "minority group".ti,ab OR "minority groups".ti,ab OR "minority health".ti,ab OR "Poverty".ti,ab OR "SES".ti,ab OR "Social Class".ti,ab OR "Social Classes".ti,ab OR "socioeconomic disparities".ti,ab OR "socio-economic disparities".ti,ab OR "socioeconomic disparity".ti,ab OR "socio-economic disparity".ti,ab OR "vulnerable".ti,ab OR "vulnerables".ti,ab OR "Social Health Inequalities".ti,ab OR "Social Health Inequality".ti,ab OR "Social Inequalities".ti,ab OR "Social Inequality".ti,ab OR "Healthcare Disparities".ti,ab) AND ("computer-assisted therapy".ti OR "computer-assisted therapy".ti OR "computer assisted therapy".ti OR "computer-assisted".ti OR "computer assisted".ti OR "online therapy".ti OR computer application*.ti OR "Electronic Learning".ti OR "Computer Mediated Communication".ti OR "Computer Mediated Communications".ti OR "Internet".ti OR "internet".ti OR "Electronic mail".ti OR "electronic mail".ti OR "e mail".ti OR "e-mail".ti OR "email".ti OR "Telemedicine".ti OR "telemedicine".ti OR telemed*.ti OR "remote".ti OR "web-based".ti OR "webbased".ti OR "web based".ti OR "e-health".ti OR "e health".ti OR "ehealth".ti OR "m-health".ti OR "m health".ti OR "mhealth".ti OR "mobile health".ti OR "mobile".ti OR "online".ti OR "on-line".ti OR "on line".ti OR "digital".ti OR "telehealth".ti OR "tele-health".ti OR "tele health".ti OR "iCBT".ti OR "mobile application".ti OR "Mobile applications".ti OR "mobile applications".ti OR "mobile application".ti OR "mobile apps".ti OR "mobile app".ti OR e-consult*.ti OR econsult*.ti OR "remote communication".ti OR "remote computer".ti OR "remote computers".ti OR "remote consultation".ti OR "remote health care".ti OR "remote healthcare".ti OR "remote monitoring".ti OR "remote system".ti OR "remote systems".ti OR "remote technologies".ti OR "remote technology".ti OR "teleconsultation".ti OR teleconsult*.ti OR "smart technology".ti OR smart technol*.ti OR "wearable technology".ti OR "wearable technologies".ti OR "Telephone".ti OR telephon*.ti OR "phone".ti OR "phones".ti OR "Cell Phones".ti OR "Smartphone".ti OR "Cell Phone".ti OR "cellular phone".ti OR "cellular phones".ti OR "Smartphones".ti OR iphon*.ti OR "Text Messaging".ti OR text messag*.ti OR "texting".ti OR "short message service".ti OR "SMS".ti OR "app".ti OR "apps".ti OR webapp*.ti OR "mass communication".ti OR "blogging".ti OR "blog".ti OR "weblog".ti OR "social media".ti OR twitter*.ti OR facebook*.ti OR webcast*.ti OR "Webcasts as Topic".ti OR "Telecommunications".ti OR web portal*.ti OR electronic communication*.ti OR "tele-care".ti OR "telecare".ti OR "tele-monitoring".ti OR "telemonitoring".ti OR "website".ti OR "websites".ti OR "wireless".ti OR "personal digital assistant".ti OR "computer-assisted instruction".ti OR "social network".ti OR social network*.ti OR "ipad".ti OR ipad*.ti OR "telenursing".ti OR telenurs*.ti OR "virtual community".ti OR "webpage".ti OR "webpages".ti OR "web application".ti OR "web applications".ti OR "web access".ti OR "web".ti OR "telerehabilitation".ti) AND ("Life style".ti,ab OR "life style".ti,ab OR "lifestyle".ti,ab OR "life-style".ti,ab OR "life styles".ti,ab OR "lifestyles".ti,ab OR "life-styles".ti,ab OR "Health behavior".ti,ab OR "health behaviour".ti,ab OR "health behavior".ti,ab OR "Health promotion".ti,ab OR "health promotion".ti,ab OR "self-management".ti,ab OR "self management".ti,ab OR "Exercise".ti,ab OR "exercise".ti,ab OR "physical activity".ti,ab OR "Diet".ti,ab OR "Diet Therapy".ti,ab OR "diet".ti,ab OR "diets".ti,ab OR "dietary".ti,ab OR "fat".ti,ab OR "salt".ti,ab OR "natrium".ti,ab OR "sodium".ti,ab OR "Dietary Carbohydrates".ti,ab OR carbohydrate*.ti,ab OR "calories".ti,ab OR "Dietary Proteins".ti,ab OR "proteins".ti,ab OR "fat intake".ti,ab OR "salt intake".ti,ab OR "Eating".ti,ab OR "eating".ti,ab OR "Nutrition Therapy".ti,ab OR "nutrition".ti,ab OR "Smoking".ti,ab OR "smoking".ti,ab OR "Tobacco use".ti,ab OR "tobacco".ti,ab OR "nicotine".ti,ab OR "cigarettes".ti,ab OR "cigarette".ti,ab OR "Drinking Behavior".ti,ab OR "alcohol drinking".ti,ab OR "Alcohol Abstinence".ti,ab OR "Alcohol Drinking".ti,ab OR "alcohol".ti,ab OR "drinking".ti,ab OR "drinking".ti,ab OR "Sleep".ti,ab OR "sleep".ti,ab OR "sedentary".ti,ab OR "Medication Adherence".ti,ab OR "Medication Adherence".ti,ab OR "medication".ti,ab OR "Body Weight".ti,ab OR "weight".ti,ab OR "body weight".ti,ab OR "Blood glucose".ti,ab OR "blood glucose".ti,ab OR "BMI".ti,ab OR "Body mass index".ti,ab OR "body mass index".ti,ab OR "overweight".ti,ab OR "overweight".ti,ab OR "obesity".ti,ab OR "obesity".ti,ab OR "obese".ti,ab OR "Blood pressure".ti,ab OR "Hypertension".ti,ab OR "blood pressure".ti,ab OR "blood pressure determination".ti,ab OR "Cholesterol".ti,ab OR "cholesterol".ti,ab OR "triglycerides".ti,ab OR "triglycerides".ti,ab OR "triglyceride".ti,ab OR "glycated hemoglobin A".ti,ab OR "hemoglobin A1c".ti,ab OR "HbA1c".ti,ab OR "glomerular filtration rate".ti,ab OR "GFR".ti,ab OR "glucoregulation".ti,ab OR "cardiac rehabilitation".ti,ab) AND ("reach".ti,ab OR reach*.ti,ab OR "outreach".ti,ab OR outreach*.ti,ab OR "Community-Institutional Relations".ti,ab OR "use".ti,ab OR "uses".ti,ab OR "used".ti,ab OR "utilise".ti,ab OR "utilize".ti,ab OR utilis*.ti,ab OR utiliz*.ti,ab OR "usage".ti,ab OR usage*.ti,ab OR "Patient Participation".ti,ab OR "implementation".ti,ab OR implement*.ti,ab OR "Health Plan Implementation".ti,ab OR "Implementation Science".ti,ab OR "Marketing".ti,ab OR "Public Relations".ti,ab OR "Program Evaluation".ti,ab OR "development".ti,ab OR "developing".ti,ab OR "develop".ti,ab OR "uptake".ti,ab) NOT (("Infant".ti OR "Child".ti OR "Adolescent".ti OR "Infant".ti OR "infants".ti OR "Child".ti OR "children".ti OR pediatr*.ti OR paediatr*.ti OR Adolescen*.ti OR "kids".ti OR "Childhood".ti OR "youth".ti OR "youths".ti) NOT "Adult".ti) NOT (conference review or conference abstract).pt)

**Web of Science**

(ti=("Minority Groups" OR "Minority Health" OR "Poverty" **OR** "deprived communities" OR "deprived community" OR "disadvantaged communities" OR "disadvantaged community" OR "disadvantaged families" OR "disadvantaged family" OR "Economic Status" OR "high-risk families" OR "high-risk family" OR "low educated" OR "low education" OR "low educational" OR "low income" OR "low incomes" OR "low social" OR "low socio" OR "low socio economic" OR "lower educated" OR "lower education" OR "lower educational" OR "lower income" OR "lower incomes" OR "lower social" OR "lower socio economic" OR "lowest educated" OR "lowest education" OR "lowest educational" OR "lowest income" OR "lowest incomes" OR "lowest social" OR "minority communities" OR "minority community" OR "minority group" OR "minority groups" OR "minority health" OR "Poverty" OR "SES" OR "Social Class" OR "Social Classes" OR "socioeconomic disparities" OR "socio-economic disparities" OR "socioeconomic disparity" OR "socio-economic disparity" OR "vulnerable" OR "vulnerables" OR "Social Health Inequalities" OR "Social Health Inequality" OR "Social Inequalities" OR "Social Inequality" OR "Healthcare Disparities" OR "Poverty" OR "deprived communities" OR "deprived community" OR "disadvantaged communities" OR "disadvantaged community" OR "disadvantaged families" OR "disadvantaged family" OR "Economic Status" OR "high-risk families" OR "high-risk family" OR "low educated" OR "low education" OR "low educational" OR "low income" OR "low incomes" OR "low social" OR "low socio" OR "low socio economic" OR "lower educated" OR "lower education" OR "lower educational" OR "lower income" OR "lower incomes" OR "lower social" OR "lower socio economic" OR "lowest educated" OR "lowest education" OR "lowest educational" OR "lowest income" OR "lowest incomes" OR "lowest social" OR "minority communities" OR "minority community" OR "minority group" OR "minority groups" OR "minority health" OR "Poverty" OR "SES" OR "Social Class" OR "Social Classes" OR "socioeconomic disparities" OR "socio-economic disparities" OR "socioeconomic disparity" OR "socio-economic disparity" OR "vulnerable" OR "vulnerables" OR "Social Health Inequalities" OR "Social Health Inequality" OR "Social Inequalities" OR "Social Inequality" OR "Healthcare Disparities") AND ti=("computer-assisted therapy" OR "computer-assisted therapy" OR "computer assisted therapy" OR "computer-assisted" OR "computer assisted" OR "online therapy" OR computer application* OR "Electronic Learning" OR "Computer Mediated Communication" OR "Computer Mediated Communications" OR "Internet" OR "internet" OR "Electronic mail" OR "electronic mail" OR "e mail" OR "e-mail" OR "email" OR "Telemedicine" OR "telemedicine" OR telemed* OR "remote" OR "web-based" OR "webbased" OR "web based" OR "e-health" OR "e health" OR "ehealth" OR "m-health" OR "m health" OR "mhealth" OR "mobile health" OR "mobile" OR "online" OR "on-line" OR "on line" OR "digital" OR "telehealth" OR "tele-health" OR "tele health" OR "iCBT" OR "mobile application" OR "Mobile applications" OR "mobile applications" OR "mobile application" OR "mobile apps" OR "mobile app" OR e-consult* OR econsult* OR "remote communication" OR "remote computer" OR "remote computers" OR "remote consultation" OR "remote health care" OR "remote healthcare" OR "remote monitoring" OR "remote system" OR "remote systems" OR "remote technologies" OR "remote technology" OR "teleconsultation" OR teleconsult* OR "smart technology" OR smart technol* OR "wearable technology" OR "wearable technologies" OR "Telephone" OR telephon* OR "phone" OR "phones" OR "Cell Phones" OR "Smartphone" OR "Cell Phone" OR "cellular phone" OR "cellular phones" OR "Smartphones" OR iphon* OR "Text Messaging" OR text messag* OR "texting" OR "short message service" OR "SMS" OR "app" OR "apps" OR webapp* OR "mass communication" OR "blogging" OR "blog" OR "weblog" OR "social media" OR twitter* OR facebook* OR webcast* OR "Webcasts as Topic" OR "Telecommunications" OR web portal* OR electronic communication* OR "tele-care" OR "telecare" OR "tele-monitoring" OR "telemonitoring" OR "website" OR "websites" OR "wireless" OR "personal digital assistant" OR "computer-assisted instruction" OR "social network" OR social network* OR "ipad" OR ipad* OR "telenursing" OR telenurs* OR "virtual community" OR "webpage" OR "webpages" OR "web application" OR "web applications" OR "web access" OR "web" OR "telerehabilitation") AND ts=("Life style" OR "life style" OR "lifestyle" OR "life-style" OR "life styles" OR "lifestyles" OR "life-styles" OR "Health behavior" OR "health behaviour" OR "health behavior" OR "Health promotion" OR "health promotion" OR "self-management" OR "self management" OR "Exercise" OR "exercise" OR "physical activity" OR "Diet" OR "Diet Therapy" OR "diet" OR "diets" OR "dietary" OR "fat" OR "salt" OR "natrium" OR "sodium" OR "Dietary Carbohydrates" OR carbohydrate* OR "calories" OR "Dietary Proteins" OR "proteins" OR "fat intake" OR "salt intake" OR "Eating" OR "eating" OR "Nutrition Therapy" OR "nutrition" OR "Smoking" OR "smoking" OR "Tobacco use" OR "tobacco" OR "nicotine" OR "cigarettes" OR "cigarette" OR "Drinking Behavior" OR "alcohol drinking" OR "Alcohol Abstinence" OR "Alcohol Drinking" OR "alcohol" OR "drinking" OR "drinking" OR "Sleep" OR "sleep" OR "sedentary" OR "Medication Adherence" OR "Medication Adherence" OR "medication" OR "Body Weight" OR "weight" OR "body weight" OR "Blood glucose" OR "blood glucose" OR "BMI" OR "Body mass index" OR "body mass index" OR "overweight" OR "overweight" OR "obesity" OR "obesity" OR "obese" OR "Blood pressure" OR "Hypertension" OR "blood pressure" OR "blood pressure determination" OR "Cholesterol" OR "cholesterol" OR "triglycerides" OR "triglycerides" OR "triglyceride" OR "glycated hemoglobin A" OR "hemoglobin A1c" OR "HbA1c" OR "glomerular filtration rate" OR "GFR" OR "glucoregulation" OR "cardiac rehabilitation") AND ts=("reach" OR reach* OR "outreach" OR outreach* OR "Community-Institutional Relations" OR "use" OR "uses" OR "used" OR "utilise" OR "utilize" OR utilis* OR utiliz* OR "usage" OR usage* OR "Patient Participation" OR "implementation" OR implement* OR "Health Plan Implementation" OR "Implementation Science" OR "Marketing" OR "Public Relations" OR "Program Evaluation" OR "development" OR "developing" OR "develop" OR "uptake") NOT ti=(("Infant" OR "Child" OR "Adolescent" OR "Infant" OR "infants" OR "Child" OR "children" OR pediatr* OR paediatr* OR Adolescen* OR "kids" OR "Childhood" OR "youth" OR "youths") NOT "Adult")) **OR** (ts=("Minority Groups" OR "Minority Health" OR "Poverty" **OR** "deprived communities" OR "deprived community" OR "disadvantaged communities" OR "disadvantaged community" OR "disadvantaged families" OR "disadvantaged family" OR "Economic Status" OR "high-risk families" OR "high-risk family" OR "low educated" OR "low education" OR "low educational" OR "low income" OR "low incomes" OR "low social" OR "low socio" OR "low socio economic" OR "lower educated" OR "lower education" OR "lower educational" OR "lower income" OR "lower incomes" OR "lower social" OR "lower socio economic" OR "lowest educated" OR "lowest education" OR "lowest educational" OR "lowest income" OR "lowest incomes" OR "lowest social" OR "minority communities" OR "minority community" OR "minority group" OR "minority groups" OR "minority health" OR "Poverty" OR "SES" OR "Social Class" OR "Social Classes" OR "socioeconomic disparities" OR "socio-economic disparities" OR "socioeconomic disparity" OR "socio-economic disparity" OR "vulnerable" OR "vulnerables" OR "Social Health Inequalities" OR "Social Health Inequality" OR "Social Inequalities" OR "Social Inequality" OR "Healthcare Disparities" OR "Poverty" OR "deprived communities" OR "deprived community" OR "disadvantaged communities" OR "disadvantaged community" OR "disadvantaged families" OR "disadvantaged family" OR "Economic Status" OR "high-risk families" OR "high-risk family" OR "low educated" OR "low education" OR "low educational" OR "low income" OR "low incomes" OR "low social" OR "low socio" OR "low socio economic" OR "lower educated" OR "lower education" OR "lower educational" OR "lower income" OR "lower incomes" OR "lower social" OR "lower socio economic" OR "lowest educated" OR "lowest education" OR "lowest educational" OR "lowest income" OR "lowest incomes" OR "lowest social" OR "minority communities" OR "minority community" OR "minority group" OR "minority groups" OR "minority health" OR "Poverty" OR "SES" OR "Social Class" OR "Social Classes" OR "socioeconomic disparities" OR "socio-economic disparities" OR "socioeconomic disparity" OR "socio-economic disparity" OR "vulnerable" OR "vulnerables" OR "Social Health Inequalities" OR "Social Health Inequality" OR "Social Inequalities" OR "Social Inequality" OR "Healthcare Disparities") AND ti=("computer-assisted therapy" OR "computer-assisted therapy" OR "computer assisted therapy" OR "computer-assisted" OR "computer assisted" OR "online therapy" OR computer application* OR "Electronic Learning" OR "Computer Mediated Communication" OR "Computer Mediated Communications" OR "Internet" OR "internet" OR "Electronic mail" OR "electronic mail" OR "e mail" OR "e-mail" OR "email" OR "Telemedicine" OR "telemedicine" OR telemed* OR "remote" OR "web-based" OR "webbased" OR "web based" OR "e-health" OR "e health" OR "ehealth" OR "m-health" OR "m health" OR "mhealth" OR "mobile health" OR "mobile" OR "online" OR "on-line" OR "on line" OR "digital" OR "telehealth" OR "tele-health" OR "tele health" OR "iCBT" OR "mobile application" OR "Mobile applications" OR "mobile applications" OR "mobile application" OR "mobile apps" OR "mobile app" OR e-consult* OR econsult* OR "remote communication" OR "remote computer" OR "remote computers" OR "remote consultation" OR "remote health care" OR "remote healthcare" OR "remote monitoring" OR "remote system" OR "remote systems" OR "remote technologies" OR "remote technology" OR "teleconsultation" OR teleconsult* OR "smart technology" OR smart technol* OR "wearable technology" OR "wearable technologies" OR "Telephone" OR telephon* OR "phone" OR "phones" OR "Cell Phones" OR "Smartphone" OR "Cell Phone" OR "cellular phone" OR "cellular phones" OR "Smartphones" OR iphon* OR "Text Messaging" OR text messag* OR "texting" OR "short message service" OR "SMS" OR "app" OR "apps" OR webapp* OR "mass communication" OR "blogging" OR "blog" OR "weblog" OR "social media" OR twitter* OR facebook* OR webcast* OR "Webcasts as Topic" OR "Telecommunications" OR web portal* OR electronic communication* OR "tele-care" OR "telecare" OR "tele-monitoring" OR "telemonitoring" OR "website" OR "websites" OR "wireless" OR "personal digital assistant" OR "computer-assisted instruction" OR "social network" OR social network* OR "ipad" OR ipad* OR "telenursing" OR telenurs* OR "virtual community" OR "webpage" OR "webpages" OR "web application" OR "web applications" OR "web access" OR "web" OR "telerehabilitation") AND ti=("Life style" OR "life style" OR "lifestyle" OR "life-style" OR "life styles" OR "lifestyles" OR "life-styles" OR "Health behavior" OR "health behaviour" OR "health behavior" OR "Health promotion" OR "health promotion" OR "self-management" OR "self management" OR "Exercise" OR "exercise" OR "physical activity" OR "Diet" OR "Diet Therapy" OR "diet" OR "diets" OR "dietary" OR "fat" OR "salt" OR "natrium" OR "sodium" OR "Dietary Carbohydrates" OR carbohydrate* OR "calories" OR "Dietary Proteins" OR "proteins" OR "fat intake" OR "salt intake" OR "Eating" OR "eating" OR "Nutrition Therapy" OR "nutrition" OR "Smoking" OR "smoking" OR "Tobacco use" OR "tobacco" OR "nicotine" OR "cigarettes" OR "cigarette" OR "Drinking Behavior" OR "alcohol drinking" OR "Alcohol Abstinence" OR "Alcohol Drinking" OR "alcohol" OR "drinking" OR "drinking" OR "Sleep" OR "sleep" OR "sedentary" OR "Medication Adherence" OR "Medication Adherence" OR "medication" OR "Body Weight" OR "weight" OR "body weight" OR "Blood glucose" OR "blood glucose" OR "BMI" OR "Body mass index" OR "body mass index" OR "overweight" OR "overweight" OR "obesity" OR "obesity" OR "obese" OR "Blood pressure" OR "Hypertension" OR "blood pressure" OR "blood pressure determination" OR "Cholesterol" OR "cholesterol" OR "triglycerides" OR "triglycerides" OR "triglyceride" OR "glycated hemoglobin A" OR "hemoglobin A1c" OR "HbA1c" OR "glomerular filtration rate" OR "GFR" OR "glucoregulation" OR "cardiac rehabilitation") AND ts=("reach" OR reach* OR "outreach" OR outreach* OR "Community-Institutional Relations" OR "use" OR "uses" OR "used" OR "utilise" OR "utilize" OR utilis* OR utiliz* OR "usage" OR usage* OR "Patient Participation" OR "implementation" OR implement* OR "Health Plan Implementation" OR "Implementation Science" OR "Marketing" OR "Public Relations" OR "Program Evaluation" OR "development" OR "developing" OR "develop" OR "uptake") NOT ti=(("Infant" OR "Child" OR "Adolescent" OR "Infant" OR "infants" OR "Child" OR "children" OR pediatr* OR paediatr* OR Adolescen* OR "kids" OR "Childhood" OR "youth" OR "youths") NOT "Adult"))

NOT (conference review or conference abstract).pt)

**NOT ti=("veterinary" OR "rabbit" OR "rabbits" OR "animal" OR "animals" OR "mouse" OR "mice" OR "rodent" OR "rodents" OR "rat" OR "rats" OR "pig" OR "pigs" OR "porcine" OR "horse" OR "horses" OR "equine" OR "cow" OR "cows" OR "bovine" OR "goat" OR "goats" OR "sheep" OR "ovine" OR "canine" OR "dog" OR "dogs" OR "feline" OR "cat" OR "cats"))**

**Cochrane**

(("Minority Groups" OR "Minority Health" OR "Poverty" **OR** "deprived communities" OR "deprived community" OR "disadvantaged communities" OR "disadvantaged community" OR "disadvantaged families" OR "disadvantaged family" OR "Economic Status" OR "high-risk families" OR "high-risk family" OR "low educated" OR "low education" OR "low educational" OR "low income" OR "low incomes" OR "low social" OR "low socio" OR "low socio economic" OR "lower educated" OR "lower education" OR "lower educational" OR "lower income" OR "lower incomes" OR "lower social" OR "lower socio economic" OR "lowest educated" OR "lowest education" OR "lowest educational" OR "lowest income" OR "lowest incomes" OR "lowest social" OR "minority communities" OR "minority community" OR "minority group" OR "minority groups" OR "minority health" OR "Poverty" OR "SES" OR "Social Class" OR "Social Classes" OR "socioeconomic disparities" OR "socio-economic disparities" OR "socioeconomic disparity" OR "socio-economic disparity" OR "vulnerable" OR "vulnerables" OR "Social Health Inequalities" OR "Social Health Inequality" OR "Social Inequalities" OR "Social Inequality" OR "Healthcare Disparities" OR "Poverty" OR "deprived communities" OR "deprived community" OR "disadvantaged communities" OR "disadvantaged community" OR "disadvantaged families" OR "disadvantaged family" OR "Economic Status" OR "high-risk families" OR "high-risk family" OR "low educated" OR "low education" OR "low educational" OR "low income" OR "low incomes" OR "low social" OR "low socio" OR "low socio economic" OR "lower educated" OR "lower education" OR "lower educational" OR "lower income" OR "lower incomes" OR "lower social" OR "lower socio economic" OR "lowest educated" OR "lowest education" OR "lowest educational" OR "lowest income" OR "lowest incomes" OR "lowest social" OR "minority communities" OR "minority community" OR "minority group" OR "minority groups" OR "minority health" OR "Poverty" OR "SES" OR "Social Class" OR "Social Classes" OR "socioeconomic disparities" OR "socio-economic disparities" OR "socioeconomic disparity" OR "socio-economic disparity" OR "vulnerable" OR "vulnerables" OR "Social Health Inequalities" OR "Social Health Inequality" OR "Social Inequalities" OR "Social Inequality" OR "Healthcare Disparities"):ti,ab,kw AND ("computer-assisted therapy" OR "computer-assisted therapy" OR "computer assisted therapy" OR "computer-assisted" OR "computer assisted" OR "online therapy" OR computer application* OR "Electronic Learning" OR "Computer Mediated Communication" OR "Computer Mediated Communications" OR "Internet" OR "internet" OR "Electronic mail" OR "electronic mail" OR "e mail" OR "e-mail" OR "email" OR "Telemedicine" OR "telemedicine" OR telemed* OR "remote" OR "web-based" OR "webbased" OR "web based" OR "e-health" OR "e health" OR "ehealth" OR "m-health" OR "m health" OR "mhealth" OR "mobile health" OR "mobile" OR "online" OR "on-line" OR "on line" OR "digital" OR "telehealth" OR "tele-health" OR "tele health" OR "iCBT" OR "mobile application" OR "Mobile applications" OR "mobile applications" OR "mobile application" OR "mobile apps" OR "mobile app" OR e-consult* OR econsult* OR "remote communication" OR "remote computer" OR "remote computers" OR "remote consultation" OR "remote health care" OR "remote healthcare" OR "remote monitoring" OR "remote system" OR "remote systems" OR "remote technologies" OR "remote technology" OR "teleconsultation" OR teleconsult* OR "smart technology" OR smart technol* OR "wearable technology" OR "wearable technologies" OR "Telephone" OR telephon* OR "phone" OR "phones" OR "Cell Phones" OR "Smartphone" OR "Cell Phone" OR "cellular phone" OR "cellular phones" OR "Smartphones" OR iphon* OR "Text Messaging" OR text messag* OR "texting" OR "short message service" OR "SMS" OR "app" OR "apps" OR webapp* OR "mass communication" OR "blogging" OR "blog" OR "weblog" OR "social media" OR twitter* OR facebook* OR webcast* OR "Webcasts as Topic" OR "Telecommunications" OR web portal* OR electronic communication* OR "tele-care" OR "telecare" OR "tele-monitoring" OR "telemonitoring" OR "website" OR "websites" OR "wireless" OR "personal digital assistant" OR "computer-assisted instruction" OR "social network" OR social network* OR "ipad" OR ipad* OR "telenursing" OR telenurs* OR "virtual community" OR "webpage" OR "webpages" OR "web application" OR "web applications" OR "web access" OR "web" OR "telerehabilitation"):ti AND ("Life style" OR "life style" OR "lifestyle" OR "life-style" OR "life styles" OR "lifestyles" OR "life-styles" OR "Health behavior" OR "health behaviour" OR "health behavior" OR "Health promotion" OR "health promotion" OR "self-management" OR "self management" OR "Exercise" OR "exercise" OR "physical activity" OR "Diet" OR "Diet Therapy" OR "diet" OR "diets" OR "dietary" OR "fat" OR "salt" OR "natrium" OR "sodium" OR "Dietary Carbohydrates" OR carbohydrate* OR "calories" OR "Dietary Proteins" OR "proteins" OR "fat intake" OR "salt intake" OR "Eating" OR "eating" OR "Nutrition Therapy" OR "nutrition" OR "Smoking" OR "smoking" OR "Tobacco use" OR "tobacco" OR "nicotine" OR "cigarettes" OR "cigarette" OR "Drinking Behavior" OR "alcohol drinking" OR "Alcohol Abstinence" OR "Alcohol Drinking" OR "alcohol" OR "drinking" OR "drinking" OR "Sleep" OR "sleep" OR "sedentary" OR "Medication Adherence" OR "Medication Adherence" OR "medication" OR "Body Weight" OR "weight" OR "body weight" OR "Blood glucose" OR "blood glucose" OR "BMI" OR "Body mass index" OR "body mass index" OR "overweight" OR "overweight" OR "obesity" OR "obesity" OR "obese" OR "Blood pressure" OR "Hypertension" OR "blood pressure" OR "blood pressure determination" OR "Cholesterol" OR "cholesterol" OR "triglycerides" OR "triglycerides" OR "triglyceride" OR "glycated hemoglobin A" OR "hemoglobin A1c" OR "HbA1c" OR "glomerular filtration rate" OR "GFR" OR "glucoregulation" OR "cardiac rehabilitation"):ti,ab,kw AND ("reach" OR reach* OR "outreach" OR outreach* OR "Community-Institutional Relations" OR "use" OR "uses" OR "used" OR "utilise" OR "utilize" OR utilis* OR utiliz* OR "usage" OR usage* OR "Patient Participation" OR "implementation" OR implement* OR "Health Plan Implementation" OR "Implementation Science" OR "Marketing" OR "Public Relations" OR "Program Evaluation" OR "development" OR "developing" OR "develop" OR "uptake"):ti,ab,kw NOT (("Infant" OR "Child" OR "Adolescent" OR "Infant" OR "infants" OR "Child" OR "children" OR pediatr* OR paediatr* OR Adolescen* OR "kids" OR "Childhood" OR "youth" OR "youths") NOT "Adult"):ti) **OR** (("Minority Groups" OR "Minority Health" OR "Poverty" **OR** "deprived communities" OR "deprived community" OR "disadvantaged communities" OR "disadvantaged community" OR "disadvantaged families" OR "disadvantaged family" OR "Economic Status" OR "high-risk families" OR "high-risk family" OR "low educated" OR "low education" OR "low educational" OR "low income" OR "low incomes" OR "low social" OR "low socio" OR "low socio economic" OR "lower educated" OR "lower education" OR "lower educational" OR "lower income" OR "lower incomes" OR "lower social" OR "lower socio economic" OR "lowest educated" OR "lowest education" OR "lowest educational" OR "lowest income" OR "lowest incomes" OR "lowest social" OR "minority communities" OR "minority community" OR "minority group" OR "minority groups" OR "minority health" OR "Poverty" OR "SES" OR "Social Class" OR "Social Classes" OR "socioeconomic disparities" OR "socio-economic disparities" OR "socioeconomic disparity" OR "socio-economic disparity" OR "vulnerable" OR "vulnerables" OR "Social Health Inequalities" OR "Social Health Inequality" OR "Social Inequalities" OR "Social Inequality" OR "Healthcare Disparities" OR "Poverty" OR "deprived communities" OR "deprived community" OR "disadvantaged communities" OR "disadvantaged community" OR "disadvantaged families" OR "disadvantaged family" OR "Economic Status" OR "high-risk families" OR "high-risk family" OR "low educated" OR "low education" OR "low educational" OR "low income" OR "low incomes" OR "low social" OR "low socio" OR "low socio economic" OR "lower educated" OR "lower education" OR "lower educational" OR "lower income" OR "lower incomes" OR "lower social" OR "lower socio economic" OR "lowest educated" OR "lowest education" OR "lowest educational" OR "lowest income" OR "lowest incomes" OR "lowest social" OR "minority communities" OR "minority community" OR "minority group" OR "minority groups" OR "minority health" OR "Poverty" OR "SES" OR "Social Class" OR "Social Classes" OR "socioeconomic disparities" OR "socio-economic disparities" OR "socioeconomic disparity" OR "socio-economic disparity" OR "vulnerable" OR "vulnerables" OR "Social Health Inequalities" OR "Social Health Inequality" OR "Social Inequalities" OR "Social Inequality" OR "Healthcare Disparities"):ti AND ("computer-assisted therapy" OR "computer-assisted therapy" OR "computer assisted therapy" OR "computer-assisted" OR "computer assisted" OR "online therapy" OR computer application* OR "Electronic Learning" OR "Computer Mediated Communication" OR "Computer Mediated Communications" OR "Internet" OR "internet" OR "Electronic mail" OR "electronic mail" OR "e mail" OR "e-mail" OR "email" OR "Telemedicine" OR "telemedicine" OR telemed* OR "remote" OR "web-based" OR "webbased" OR "web based" OR "e-health" OR "e health" OR "ehealth" OR "m-health" OR "m health" OR "mhealth" OR "mobile health" OR "mobile" OR "online" OR "on-line" OR "on line" OR "digital" OR "telehealth" OR "tele-health" OR "tele health" OR "iCBT" OR "mobile application" OR "Mobile applications" OR "mobile applications" OR "mobile application" OR "mobile apps" OR "mobile app" OR e-consult* OR econsult* OR "remote communication" OR "remote computer" OR "remote computers" OR "remote consultation" OR "remote health care" OR "remote healthcare" OR "remote monitoring" OR "remote system" OR "remote systems" OR "remote technologies" OR "remote technology" OR "teleconsultation" OR teleconsult* OR "smart technology" OR smart technol* OR "wearable technology" OR "wearable technologies" OR "Telephone" OR telephon* OR "phone" OR "phones" OR "Cell Phones" OR "Smartphone" OR "Cell Phone" OR "cellular phone" OR "cellular phones" OR "Smartphones" OR iphon* OR "Text Messaging" OR text messag* OR "texting" OR "short message service" OR "SMS" OR "app" OR "apps" OR webapp* OR "mass communication" OR "blogging" OR "blog" OR "weblog" OR "social media" OR twitter* OR facebook* OR webcast* OR "Webcasts as Topic" OR "Telecommunications" OR web portal* OR electronic communication* OR "tele-care" OR "telecare" OR "tele-monitoring" OR "telemonitoring" OR "website" OR "websites" OR "wireless" OR "personal digital assistant" OR "computer-assisted instruction" OR "social network" OR social network* OR "ipad" OR ipad* OR "telenursing" OR telenurs* OR "virtual community" OR "webpage" OR "webpages" OR "web application" OR "web applications" OR "web access" OR "web" OR "telerehabilitation"):ti,ab,kw AND ("Life style" OR "life style" OR "lifestyle" OR "life-style" OR "life styles" OR "lifestyles" OR "life-styles" OR "Health behavior" OR "health behaviour" OR "health behavior" OR "Health promotion" OR "health promotion" OR "self-management" OR "self management" OR "Exercise" OR "exercise" OR "physical activity" OR "Diet" OR "Diet Therapy" OR "diet" OR "diets" OR "dietary" OR "fat" OR "salt" OR "natrium" OR "sodium" OR "Dietary Carbohydrates" OR carbohydrate* OR "calories" OR "Dietary Proteins" OR "proteins" OR "fat intake" OR "salt intake" OR "Eating" OR "eating" OR "Nutrition Therapy" OR "nutrition" OR "Smoking" OR "smoking" OR "Tobacco use" OR "tobacco" OR "nicotine" OR "cigarettes" OR "cigarette" OR "Drinking Behavior" OR "alcohol drinking" OR "Alcohol Abstinence" OR "Alcohol Drinking" OR "alcohol" OR "drinking" OR "drinking" OR "Sleep" OR "sleep" OR "sedentary" OR "Medication Adherence" OR "Medication Adherence" OR "medication" OR "Body Weight" OR "weight" OR "body weight" OR "Blood glucose" OR "blood glucose" OR "BMI" OR "Body mass index" OR "body mass index" OR "overweight" OR "overweight" OR "obesity" OR "obesity" OR "obese" OR "Blood pressure" OR "Hypertension" OR "blood pressure" OR "blood pressure determination" OR "Cholesterol" OR "cholesterol" OR "triglycerides" OR "triglycerides" OR "triglyceride" OR "glycated hemoglobin A" OR "hemoglobin A1c" OR "HbA1c" OR "glomerular filtration rate" OR "GFR" OR "glucoregulation" OR "cardiac rehabilitation"):ti,ab,kw AND ("reach" OR reach* OR "outreach" OR outreach* OR "Community-Institutional Relations" OR "use" OR "uses" OR "used" OR "utilise" OR "utilize" OR utilis* OR utiliz* OR "usage" OR usage* OR "Patient Participation" OR "implementation" OR implement* OR "Health Plan Implementation" OR "Implementation Science" OR "Marketing" OR "Public Relations" OR "Program Evaluation" OR "development" OR "developing" OR "develop" OR "uptake"):ti,ab,kw NOT (("Infant" OR "Child" OR "Adolescent" OR "Infant" OR "infants" OR "Child" OR "children" OR pediatr* OR paediatr* OR Adolescen* OR "kids" OR "Childhood" OR "youth" OR "youths") NOT "Adult"):ti)
