# Supplementary material for: The Barriers and Facilitators of eHealth-Based Lifestyle Intervention Programs for People With a Low Socioeconomic Status: Scoping Review
Source: J Med Internet Res. 2022 Aug 24;24(8):e34229. doi: 10.2196/34229 (PMC9453585; doi:10.2196/34229)
Supplement: Multimedia Appendix 2 [file jmir_v24i8e34229_app2.doc]

Multi-media Appendix 2– Key constructs and definitions for data extraction

**(Behavioral) theory** = A theoretical background to guide the development of eHealth intervention

**Development:** is a process to develop and design the intervention in which the actual functioning of eHealth technology is created via prototyping, usability testing and the addition of persuasive elements and behavior change techniques.

**eHealth** = The use of technology to support health, well-being, and healthcare.

**Evaluation =** Activities throughout the entire development process that provide ongoing information on improving the development process, outcomes of activities and eHealth technology.

**Implementation** = It refers to the intervention agents’ fidelity to the various elements of an intervention’s key functions or components, including consistency of delivery as intended and the time and cost of the intervention. It also includes adaptations made to interventions and implementation

strategies.

**Lifestyle** = Health-related lifestyle component (physical activity, diet, alcohol, smoking, sleep, overweight)

**Socioeconomic status** = position of an individual on a socioeconomic scale that measures factors by a single variable at a single level, such as education, income, or neighborhood status

**Reach** = The absolute number, proportion, and representativeness of individuals who are willing to participate in a given initiative, intervention, or program, and reasons why or why not.

**Use** = Intended usage: the extent to which the developers of the intervention felt that the intervention should be used to achieve the desired effect; Actual Usage: information about the usage of the intervention can be collected, for example, the number of times the user or patient logged on and the number of modules completed.

**User** = The person who actively interacts with the system.

## References

Glasgow RE, Harden SM, Gaglio B, Rabin B, Smith ML, Porter GC, Ory MG, Estabrooks PA. RE-AIM planning and evaluation framework: Adapting to new science and practice with a 20-year review. Front Public Heal 2019;7(MAR). [doi: 10.3389/fpubh.2019.00064]

van Gemert-Pijnen LJ, Kelders SM, Kip H, Sanderman R, editors. eHealth research, theory and development: a multi-disciplinary approach. Abingdon-on-Thames, UK: Routledge; 2018:331-341.

Kip H, van Gemert-Pijnen LJ. Holistic development of eHealth technology. In: van Gemert-Pijnen LJ, Kelders SM, Kip H, Sanderman R, editors. eHealth Research, Theory and Development: A Multi-Disciplinary Approach. Abingdon-on-Thames, UK: Routledge; 2018:131-186
